# Questions and answers in the management of children with medulloblastoma over the time. How did we get here? A systematic review

**DOI:** 10.3389/fonc.2023.1229853

**Published:** 2023-06-29

**Authors:** Marta P. Osuna-Marco, Laura I. Martín-López, Águeda M. Tejera, Blanca López-Ibor

**Affiliations:** ^1^ Pediatric Oncology Unit, Centro Integral Oncológico Clara Campal (CIOCC), Hospital Universitario HM Montepríncipe, HM Hospitales, Madrid, Spain; ^2^ Faculty of Experimental Sciences, Universidad Francisco de Vitoria, Madrid, Spain

**Keywords:** Medulloblastoma, children, survival, surgery, radiotherapy, chemotherapy, cancer treatment protocol, side effects

## Abstract

**Introduction:**

Treatment of children with medulloblastoma (MB) includes surgery, radiation therapy (RT) and chemotherapy (CT). Several treatment protocols and clinical trials have been developed over the time to maximize survival and minimize side effects.

**Methods:**

We performed a systematic literature search in May 2023 using PubMed. We selected all clinical trials articles and multicenter studies focusing on MB. We excluded studies focusing exclusively on infants, adults, supratentorial PNETs or refractory/relapsed tumors, studies involving different tumors or different types of PNETs without differentiating survival, studies including <10 cases of MB, solely retrospective studies and those without reference to outcome and/or side effects after a defined treatment.

**Results:**

1. The main poor-prognosis factors are: metastatic disease, anaplasia, MYC amplification, age younger than 36 months and some molecular subgroups. The postoperative residual tumor size is controversial.

2. MB is a collection of diseases.

3. MB is a curable disease at diagnosis, but survival is scarce upon relapse.

4. Children should be treated by experienced neurosurgeons and in advanced centers.

5. RT is an essential treatment for MB. It should be administered craniospinal, early and without interruptions.

6. Craniospinal RT dose could be lowered in some low-risk patients, but these reductions should be done with caution to avoid relapses.

7. Irradiation of the tumor area instead of the entire posterior fossa is safe enough.

8. Hyperfractionated RT is not superior to conventional RT

9. Both photon and proton RT are effective.

10. CT increases survival, especially in high-risk patients.

11. There are multiple drugs effective in MB. The combination of different drugs is appropriate management.

12. CT should be administered after RT.

13. The specific benefit of concomitant CT to RT is unknown.

14. Intensified CT with stem cell rescue has no benefit compared to standard CT regimens.

15. The efficacy of intraventricular/intrathecal CT is controversial.

16. We should start to think about incorporating targeted therapies in front-line treatment.

17. Survivors of MB still have significant side effects.

**Conclusion:**

Survival rates of MB improved greatly from 1940-1970, but since then the improvement has been smaller. We should consider introducing targeted therapy as front-line therapy.

## 1 Introduction

Medulloblastoma (MB) was first described in 1925 and is to date the most common malignant brain tumor of childhood (1). It is an embryonal tumor that arises in the cerebellum and is characterized by the presence of small blue round cells. It has a peak age of diagnosis around 6-8 years of age (2). Almost 25-35% of MB occur in children under 3 years old and about 50% are diagnosed before the age of 5 years old (3, 4). Since 2011, it has been classified into four molecular subgroups: WNT-MB, SHH-MB, Group 3 MB and Group 4 MB (2, 5). Progressively, different subtypes are being redefined based on further genomic findings (6). There is increasing awareness of the importance of these molecular subgroups in terms of prognosis and specific therapeutic management (7, 8).

**Figure 1:**
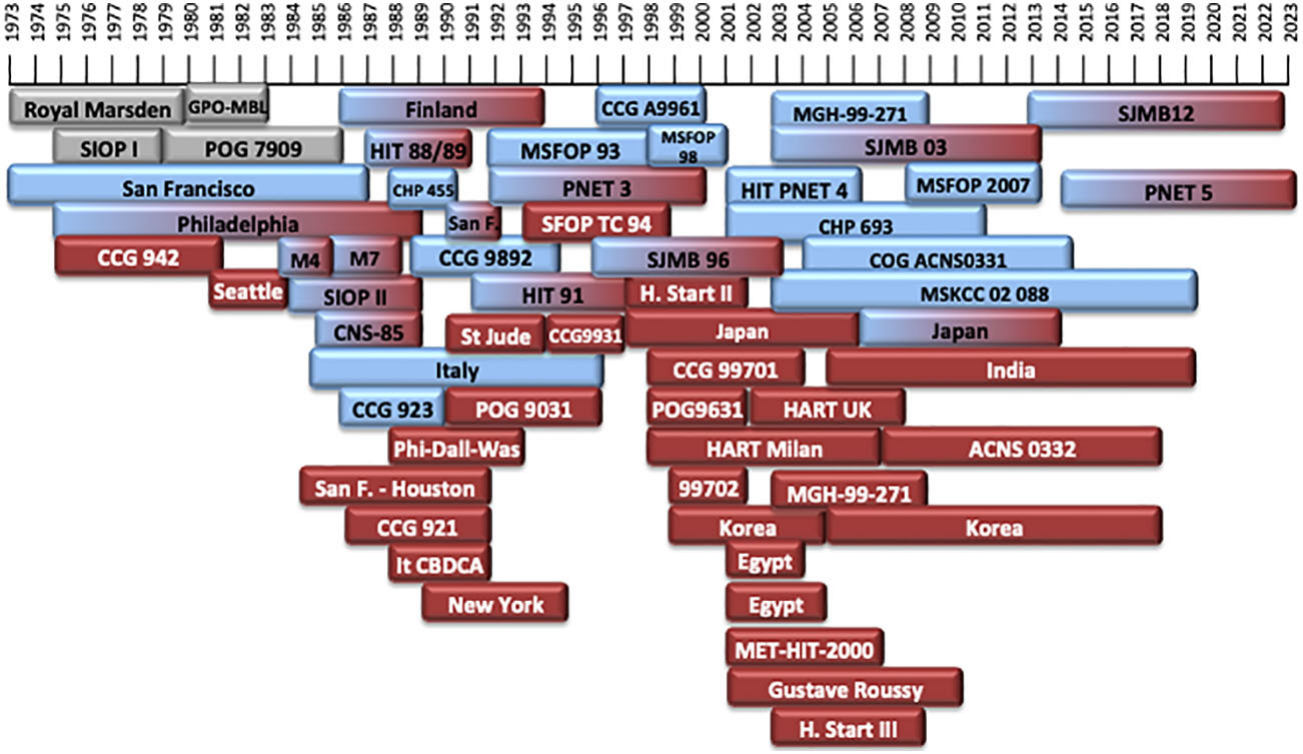
Timeline of the different treatment protocols developed over the last 50 years. In grey: treatment protocols of non-defined risk MB (NR-MB). In blue: treatment protocols for patients with average-risk MB (AR-MB). In red: treatment protocols for patients with high-risk MB (HR-MB). Protocols with gradient colors included patients both with AR-MB or HR-MB.

Treatment of MB generally includes surgery, chemotherapy (CT) and radiation therapy (RT) (2). Treatment of MB has progressed greatly over the past 70 years. The earliest treatment protocols date back to the 1960s (9) (**Tables 1**–**4**). Different treatment protocols have been designed internationally to find the best management of MB (**Figure 1**) (**Tables 1**–**4**).

**Table 1:** Protocols of treatment developed over the time for patients with MB without classification by risk.

	Trial	Dates	N (total/AR/HR)	Patient ages (years)	Treatment (chronological order)	Survival	Main conclusion of the study	Reference
Average-risk MB	Manchester	1941-1950	22	1-13	Sx + RT	3-year OS 65%	Improvement of survival	(10)
	Royal Marsden	1952-1970	87	0-15	Sx + RT&CT + CT	3-year DFS 49%; 5-year OS 32%	Adding CT is beneficial	(11)
	Indiana	1953-1973	45	1-16	Sx + RT	5-year OS 41%; 10-year OS 22%	Higher RT doses confer better OS Adding CT could be beneficial	(12)
	Denmark	1963-1975	44	1-54	Sx + RT (different doses randomized)	5-year OS 33%; 10-year OS 20%	RT is necessary. It must start as soon as possible	(13)
	London	1965-1974	87	0-11 y.o	Sx + RT + CT	5-year OS 2% (1965–1969) Vs. 39% (1970–1974)	There is an improvement in survival, probably secondary to improved RT technique	(14)
	Philadelphia-Pennsylvania	1969-1979	24	2-19	Sx + RT/RT&CT**^¶^** + CT	4-year PFS 38% (without CT and operating microscope) Vs. 84% (with CT and operating microscope)	Modern surgical techniques improve survival	(15)
	Royal Marsden	1970-1980	37	0-15	Sx + RT/RT&CT**^¶^** + CT**^¶^**	3-year DFS 62%; 5-year OS 71%	Adding CT is beneficial	(11)
	SIOP I	1975-1979	286	0-15	Sx + RT	Globally, 5-year DFS 48% and 5-year OS 53%; 5-year DFS 52% (RT+CT) Vs. 42% (RT);	Adding CT is beneficial	(16)
					Sx + RT&CT + CT			
	POG 7909	1979-1986	78	1-21	Sx + RT	5-year EFS 57% (RT); 5-year OS 56% (RT)	Adding CT is beneficial, specially in children >= 5 years old	(17)
					Sx + RT + CT	5-year EFS 68% (RT+MOPP); 5-year OS 74% (RT+MOPP)		
	GPO-MBL 80	1980-1983	69	0.75-22	Sx + CT + RT	6-year EFS 46%	“Sandwich CT” is feasible. There is no benefit in maintenance CT	(18, 19)
					Sx + CT + RT + CT			

AR, average-risk; CT, chemotherapy; DFS, disease-free survival; EFS, event-free survival; HR, high-risk; MB, medulloblastoma; MOPP, nitrogen mustard, vincristine, prednisone, and procarbazine; N, number of patients included; OS, overall survival; PFS, progression-free survival; RT, radiation therapy; RT&CT, radiation therapy with concomitant chemotherapy; Sx, surgery; ^¶^, only in some patients.

**Table 2:** Protocols of treatment developed over the time for patients with low-risk MB.

	Trial	Dates	N (total/AR/HR)	Patient ages (years)	Treatment (chronological order)	Survival	Main conclusion of the study	Reference
Low-risk MB	SJMB12 stratum W1	2013- present	No results yet	3-39	Sx + RT + CT	No results yet	No results yet	–
	SJMB12 stratum W2	2013- present	No results yet	3-39	Sx + RT + CT	No results yet	No results yet	–
	PNET5 MB-LR	2014- present	No results yet	3-21	Sx + RT + CT	No results yet	No results yet	–
	ACNS1422	2017-present	No results yet	3-21	Sx + RT + CT	No results yet	No results yet	–

AR, average risk; HR, high risk; MB, medulloblastoma; N, number of patients included; Sx, surgery.

**Table 3:** Protocols of treatment developed over the time for patients with average-risk MB.

	Trial	Dates	n (total/AR/HR)	Patient ages (years)	Treatment (chronological order)	Survival	Main conclusion of the study	Reference
Average-risk MB	New York	1963-1975	59/20/38	1-16	Sx + RT	Non specified	Improved of survival by increasing the RT dose to the PF	(20)
	San Francisco	1966-1987	65/27/ 38	1-56	Sx + RT (30-40 Gy CSI)	5-year DFS 78% (high-dose CSI); 5-year OS 91% (high-dose CSI)	Reducing the CSI dose does not result in a higher rate of tumor recurrence along in the spinal axis or in the brain	(9)
					Sx + CT (25 Gy, 36 Gy**^¶^**) + RT&CT	5-year DFS 77% (low-dose CSI + CT); 5-year OS 83% (low-dose CSI + CT)		
	CCG 942	1975-1981	233/191/42	2-16	Sx + RT	5-year EFS 62% (RT)	Adding CT is beneficial for metastatic MB	(21)
					Sx + RT&CT + CT	5-year EFS 65% (RT+CT)		
	Philadelphia	1975 - 1982	41/18/ 23	0.1-18	Sx + RT/RT&CT**^¶^** + CT**^¶^**	5-year DFS 49% (67% in standard-risk)	Adding CT is beneficial	(22)
	Philadelphia	1983 - 1989	67/20/ 47	0.5-21	Sx + RT/RT&CT**^¶^** + CT**^¶^**	5-year DFS 52% (RT alone) Vs. 88% (RT+CT)		(22)
	M4	1984-1985	16/8/8	3-17	Sx + CT + RT	6-year DFS 18% (12% in standard-risk)	Supratentorial RT is needed to avoid relapse	(25)
	SIOP II/GPOH	1984-1989	364/229/135	0-15	Sx + CT + RT (randomized 25Gy and 35Gy)	5-year EFS 58.9% (sandwich CT) Vs. 64.7% (no sandwich CT); 5-year EFS 67.6% (35 Gy) Vs. 55.3% (25 Gy)	CT prior to RT is not beneficial. Non-reduced RT dose is preferred	(26)
					Sx + RT			
	M7	1985-1988	70/31/ 37	0.83-19	Sx + CT + RT	5-year DFS 62% (74% in standard-risk)	This regimen is feasible	(28)
	CNS-85	1985-1989	38/11/ 27	0.58-14	Sx + RT	5-year EFS 47.4% (27.3% in standard-risk); 5-year OS 50% (27.3% in standard-risk);	Age is the most important prognostic factor. Adding CT could be beneficial. RT doses are important	(30)
	Italy	1985-1996	47/47/ 0	3-17	Sx + CT + RT + CT	Not specified	No differences in survival but worse cognitive outcomes in patients receiving IT MTX	(69)
	POG 8631/CCG 923	1986-1990	126/126/0	3-21	Sx + RT (23.4Gy)	5-year EFS 52% (23.4 Gy) p=0.08; 5-year OS aprox 68% (23.4 Gy) p=0.08; 17/60 relapses (23.4 Gy) at the interim analysis (Nov 1990)	Early relapse in the reduced-RT arm	(70, 71)
					Sx + RT (36 Gy)	5-year EFS 67% (36 Gy) p=0.08; 5-year OS approx 80% (36 Gy); 5/63 relapses (36 Gy) at the interim analysis (Nov 90)		
	Finland	1986-1993	39/14/ 25	0.1-16.8	Sx + CT + RT + CT	5-year PFS 59%; 5-year OS 63% (65% in standard-risk)	Better survival rates with this approach compared to historical cohort	(33)
	HIT 88/89	1987-1991	94/47/ 47	3-30	Sx + CT + RT + CT**^¶^**	5-year PFS 49% (61% in standard-risk); 5-year OS 57%	CT prior to RT is efficacious	(34)
	CH455 Philadelphia	1988-1990	10/10/ 0	1.5-5	Sx + RT&CT + CT	5-year PFS 70%; 5-year OS 70%	Reduced CSI RT dose diminishes neurocognitive damage but increases risk of relapse	(72)
	CCG 9892	1989-1994	65/65/ 0	3-10	Sx + RT&CT + CT	5-year PFS 79%; 5-year EFS 78%	Reduced-dose CSI RT combined with adjuvant CT is feasible	(73)
	San Francisco	1990-1992	25/16/ 9	3-38	Sx + HFRT	5-year PFS 49% in standard-risk; 5-year OS 69%	Disappointing results. Poor survival rates with the scheme of hyperfractionated RT.	(38)
	HIT 91	1991-1997	184/ 137/47	3-17.8	Sx + CT + RT + CT**^¶^**	3-year PFS 65% (sandwich CT); 10-year EFS 53% (sandwich CT)	Worse outcome if pre-RT CT. RT should not be delayed	(42, 43)
					Sx + RT&CT + CT	3-year PFS 78% post-RT CT), p<0.03; 10-year EFS 83% (post-RT CT),p=0.004		
	MSFOP 93	1992-1998	136/ 136/0	3-18	Sx + CT + RT	5-year PFS 73.8%, 5-year OS 64.8%	Reduced-dose CSI RT combined with CT is possible	(74)
	SIOP/UKCCSG PNET 3	1992-2000	247/ 179/68	2.91-16.79	Sx + RT	5-year EFS 59.8% (RT alone), p=0.0366; 5-year OS 64.9% (RT alone), p=0.09	Better EFS if pre-RT CT, same OS	(75)
					Sx + CT + RT	5-year EFS 74.2% (pre-RT CT + RT), p=0.0366; 5-year OS 76.7% (pre-RT CT + RT), p=0.09		
	USA Study CBDCA	prior 1997	25/ 25/0	4-21	Sx + RT&CT + CT	3-year PFS 73%	Disappointing results. CBDCA is not superior to CDDP	(76)
	Australia and New Zealand	prior 2002	19/8/ 11	0.3-9.5	Sx + CT + further treatment according to investigator preference	5-year EFS 67%; 5-year OS 67%	This CT combination is effective	(47)
	CCG A9961	1996-2000	379/ 379/ 0	3-19	Sx + RT&CT + CT (regimen CCNU+CDDP+VCR)	5-year EFS 81% globally; 5-year EFS 82% (CCNU+CDDP+VCR); 5-year OS 86% globally; 5-year OS 87% (CCNU+CDDP+VCR); 10-year EFS 75.8% globally; 10-year OS 81.3% globally.	Reduced-dose CSI RT combined with adjuvant CT is feasible. No difference between maintenance CT regimens	(77, 78)
					Sx + RT&CT + CT (CP+ CDDP+VCR)	5-year EFS 80%(CP+ CDDP+VCR); 5-year OS 85%(CP+CDDP+VCR);		
	SJMB 96	1996-2003	134/ 86/48	3.1-20.2	Sx + RT + CT	5-year EFS 83%; 5-year OS 85%.	Good outcomes despite using reduced-dose CSI RT, non-irradiation of the entire PF, and reducing total VCR and CDDP dose compared to other regimens.	(49, 79)
	MSFOP 98	1998-2001	48/48/ 0	5-18	Sx + HFRT	6-year EFS 75%, 6-year OS 78%	Hyperfractionated RT achieves very good EFS in the absence of CT	(80)
	HIT-SIOP PNET 4 (originally HIT-2000-AB4)	2001-2006	340/ 340/0	4-21	Sx + RT&CT + CT	5-year EFS 77% (standard RT), 5-year OS 87% (standard RT)	Hyperfractionated RT is not superior to conventional RT	(81)
					Sx + HFRT&CT + CT	5-year EFS 78% (hyperfractionated RT), 5-year OS 85% (hyperfractionated RT)		
	CHP693	2001-2011	28/28/ 0	3-30	Sx + RT&CT + CT	5-year PFS 71%; 5-year OS 86%	Higher risk of relapse compared to other protocols of treatment	(82)
	MGH-99-271	2003-2009	59/39/ 20	3-21	Sx + RT&CT**^¶^** + CT	5-year PFS 85%; 5-year OS 86%	Proton RT has similar outcomes and less toxicity	(62)
	SJMB03	2003-2013	330/ 227/ 103	3-21	Sx + RT + CT	5-year EFS 82.3%, 5-year PFS 83.2%, 5-year OS 88%. 5-year PFS WNT 100%, 5-year PFS SHH 77.5%, 5-year PFS group 3 66.7%, 5-year PFS group 4 87.3%	Molecular classification is essential in MB	(8)
	MSKCC 02-088	2003-2019	6-20/ 6-20/ 0	≥3	Sx + RT&CT + CT	No results yet	Results pending	–
	COG ACNS0331	2004-2014	464/ 464/0	3-21	Sx + RT&CT + CT (18Gy of CSI)	5-year EFS 18 Gy 71.4%; 5-year OS 18 Gy 77.5%; Globally: 5-year EFS WNT 93.3%, 5-year EFS SHH 82.6%, 5-year EFS group 3 63.3%, 5-year EFS group 4 86.7%	Increased risk of relapse in reduced CSI RT dose Decreasing radiation boost volume to primary site is feasible	(83)
					Sx + RT&CT + CT (23.4 Gy of CSI)	5-year EFS 23.4 Gy 82.9%; 5-year EFS 23.4 Gy 85.6%.		
					Sx + RT&CT + CT (RT to tumor bed)	5-year EFS involved-field RT 82.5%; 5-year OS involved-field RT 84.6%		
					Sx + RT&CT + CT (RT to posterior fossa)	5-year EFS posterior fossa RT 80.5%; 5-year OS posterior fossa RT 85.2%;		
	Japan 2006	2006-2014	48/35/ 13	3-11	Sx + CT + RT&CT + CT	3-year PFS 90.5%; 3-year OS 93.9%	Intensified CT including IT MTX concomitant with RT allows a reduced CSI RT dose	(66)
	MSFOP 98 & MSFOP 2007	1998-2001 2008-2013	114/ 114/0	5-18	Sx + HFRT	5-year PFS 74%; 5-year OS 84%; 5-year PFS WNT 84%, 5-year PFS SHH 67%, 5-year PFS non-WNT/non-SHH group 71%; 5-year OS WNT 95%, 5-year OS SHH 67%, 5-year OS group 3 78%, 5-year OS group 4 78%	Hyperfractionated RT achieves very good EFS in the absence of CT	(84)
	SJMB12 stratum S1 or N1	2013- present	No results yet	3-39	Sx + RT + CT	No results yet	Results pending	–
	SIOP PNET 5 MB-SR	2014- present	No results yet	3-21	Sx + RT + CT	No results yet	Results pending	–
					Sx + RT&CT + CT			

AR, average-risk; CBDCA, carboplatin; CCNU, lomustine; CDDP, cisplatin; CP, cyclophosphamide; CSI, craniospinal irradiation; CT, chemotherapy; DFS, disease-free survival; EFS, event-free survival; HR, high-risk; HFRT, hyperfractionated radiation therapy; IT, intrathecal; MB, medulloblastoma; MTX, methotrexate; N, number of patients included; OS, overall survival; PF, posterior fossa; PFS, progression-free survival; PNET, primitive neuroectodermal tumors; RT, radiation therapy; RT&CT, radiation therapy with concomitant chemotherapy; Sx, surgery; VCR, vincristine; ^¶^, only in some patients.

**Table 4:** Protocols of treatment developed over the time for patients with high-risk MB.

	Trial	Dates	n (total/AR/HR)	Patient ages (years)	Treatment (chronological order)	Survival	Main conclusion of the study	Reference
High-risk MB	New York	1963-1975	59/20/38	0-16	Sx + RT	For the 59 patients: 5-year OS 40%; 10-year OS 31%	Improved of survival by increasing the RT dose to the PF	(20)
	San Francisco	1966-1987	65/27/38	1-56	Sx + CT + RT&CT (25 Gy)	5-year DFS 39% (low-dose CSI + CT); 5-year OS 58% (low-dose CSI + CT)	Reducing the CSI dose does not result in a higher rate of tumor recurrence along in the spinal axis or in the brain	(9)
					Sx + RT (30-40 Gy)	5-year DFS 78% (high-dose CSI); 5-year OS 69% (high-dose CSI)		
	CCG 942	1975-1981	233/191/42	2-16	Sx + RT	5-year EFS 0% (RT)	Adding CT is beneficial for metastatic MB	(21)
					Sx + RT&CT + CT	5-year EFS 46% (RT+CT)		
	Philadelphia	1975-1982	41/18/23	0.1-18	Sx + RT/(RT&CT**^¶^**)+ CT**^¶^**	All 41 patients, 5-year DFS 49%. In high-risk, 5-year DFS 44%	Adding CT is beneficial	(22)
	Seattle-Columbus-Denver	1981-1984	25/0/25	unkown	Sx + CT + other treatments	2-year PFS 52.5%; 2-year OS 71.3%	This regimen is feasible	(23)
	Philadelphia	1983-1990	67/20/47	0.5-21	Sx + RT&CT + CT	All 67 patients, 5-year DFS 52% (RT alone) Vs. 88% (RT+CT) and 5-year OS 82%. In high-risk, 5-year DFS 92% (RT+CT) and 5-year OS 87%	Adding CT is beneficial	(22)
	Philadelphia, Dallas and Washington	1988-1993	63/0/63	1.5-21	Sx + RT&CT + CT	5-year PFS 85%; 5-year EFS 83%;	Adding CT is beneficial	(24)
	M4	1984-1985	16/8/8	3-17	Sx + CT + RT	6-year DFS 18% (25% in high-risk)	Supratentorial RT is needed to avoid relapse	(25)
	SIOP II/GPOH	1984-1989	364/229/135	0-15	Sx + RT + CT	5-year EFS 52.8% (no sandwich CT)	CT prior to RT is not beneficial.	(26)
					Sx + CT + RT + CT	5-year EFS 56.3% (sandwich CT)		
	San Francisco-Houston	1984-1992	27/3/24	1.9-46	Sx + CT + RT&CT + CT	5-year PFS 36%; 5-year OS 56%; For M0, 5-year PFS 52%; 5-year OS 73%; For M+, 5-year PFS 20%; 5-year OS 40%	Survival not improved. May be due to lowered CSI dose	(27)
	M7	1985-1988	70/31/37	0.83-19	Sx + CT + RT&CT + CT	5-year DFS 62% (57% in high-risk); 7-Year PFS 68% for M0; 7-rear PFS 43% for M+	This regimen is feasible. Patients with metastatic disease require intensified treatment	(28, 29)
	CNS-85	1985-1989	38/11/27	0.58-14	Sx + CT + RT&CT + CT	5-year EFS 47.4% (55.6% in high-risk); 5-year OS 50% (59.3% in high-risk);	Age is the most important prognostic factor. Adding CT could be beneficial. RT doses are important	(30)
	POG 8695	1986-1990	30/0/30	4-21	Sx + CT + RT	2-year PFS 40%; 2-year OS 61% 7/30 CR, 6/30 PR, 7/30 SD, 3/30 NR, 7/30 PD	Pre-RT CT increases relapse and toxicity	(31)
	CCG 921	1986-1992	203/0/203	1.5-21	Sx + RT&CT + CT (VCP)	Globally, 5-year PFS 54%; 5-year OS 55%; 5-year PFS 63% (VCP)	VCP is superior to “8-in-1” CT. Disease extension and, in M0 stage, residual tumor size are prognostic factors	(32)
					Sx + RT + CT (“8-in-one”)	5-year PFS 45% (8-in-1), p=0.006		
	Finland	1986-1993	39/14/25	0.1-16.8	Sx + CT + RT + CT	5-year PFS 59%; 5-year OS 63% (61% in high-risk)	Better survival rates with this approach compared to historical cohort	(33)
	HIT 88/89	1987-1991	94/47/47	3-30	Sx + CT + RT + CT**^¶^**	5-year PFS 49% (33% in high-risk); 5-year OS 57%	CT prior to RT is efficacious	(34)
	Italy CBDCA	1988-1992	13/0/13	0.8-15	Sx + CT + other treatments	1/17 CR, 7/17 PR, 4/17 SD, 1/17 PD	CBDCA is effective and safe in some patients, but ineffective in other patients.	(35)
	New York	1989-1995	23/0/23	3-25	Sx + HFRT + CT	6-year PFS 93% (M0), 3-year PFS 25% (high T and high M), 3-year PFS 0% (exocerebellar primaries)	Excellent outcome of patients with localized disease	(36)
	Rome	Unknown	12/0/12	0.42-16	Sx + CT + RT	2/8 CR, 2/8 PR, 4/8 PD	High-dose CBDCA is effective in high-risk MB, but subsequent CP has an unacceptable number of PD	(37)
	San Francisco	1990-1992	25/16/9	5-39	Sx + HFRT + CT	3-year PFS 56%	Hyperfractionated RT is not superior to standard RT	(38)
	St Jude	1990-1994	16/0/16	3.6-17.3	Sx + CT + RT + CT**^¶^**	3-year PFS 63%; 3-year OS 93%	Responses to CBDCA seem similar to other CDDP-containing regimens, but rate of progression may be slightly higher. Randomized studies are needed.	(39)
	POG 9031	1990-1996	224/0/224	3-21.4	Sx + CT + RT + CT	5-year EFS 66% (CT first), p=0.54; 5-year OS 73.1% (CT first), p=0.47	Non-statistically significant difference	(40, 41)
					Sx + RT + CT	5-year EFS 70% (RT first), p=0.54; 5-year OS 76.1% (RT first), p=0.47		
	HIT 91	1991-1997	184/137/47	3-17.8	Sx + CT + RT/(RT&CT**^¶^**) + CT**^¶^**	Globally, 3-year PFS 65% (M1); 3-year PFS 30% (M2/3) In M1 patients 10-year EFS 36% (sandwich CT) p=0.023; In M2/3 patients 10-year EFS 40% (sandwich CT), p=0.8	M1: Worse outcome if pre-RT CT M2/3: no difference in outcome	(42, 43)
					Sx + RT&CT + CT**^¶^**	In M1 patients 10-year EFS 36% 71% (post-RT CT), p=0.023; In M2/3 patients 10-year EFS 32% (post-RT CT), p=0.8		
	SIOP/UKCCSG PNET 3	1992-2000	247/179/68	2.8-16.4	Sx + CT + RT	5-year EFS 34.7% (pre-RT CT + RT); 5-year OS 43.9% (pre-RT CT + RT)	No improvement in outcome for M2/M3 patients	(44)
	SFOP TC 94	1993-1999	115/0/115	3-18	Sx + CT + RT + CT	5-year EFS 49.8%; 5-year OS 60.1%; 5-year EFS 68.8% for PF residue only; 5-year EFS 58.8% for M1; 5-year EFS 43.1% for M2/M3	Similar results. Better treatments are needed	(45)
	CCG 9931	1994-1997	124/0/124	3-22	Sx + CT + HFRT	5-year PFS 43%; 5-year OS 52%	This regimen is feasible.	(46)
	Australia and New Zealand	Unknown	19/8/11	0.3-9.5	Sx + CT + other treatments	5-year EFS 67%	This CT combination is effective	(47)
	Topotecan	Unknown	36/0/36	3.2-16.9	Sx + CT + other treatments	4/36 CR, 6/36 PR, 4/36 MR, 17/36 SD, 5/36 PD	Topotecan is an effective drug	(48)
	SJMB 96	1996-2003	134/86/48	3.1-17	Sx + CT**^¶^** + RT + CT	5-year EFS 70%; 5-year OS 70%	Promising survival rates with early high-dose RT followed by dose-intensive CT	(49)
	Head Start II	1997-2002	21/9/30	0.58-9.9	Sx + CT + RT + CT	17/21 CR, 2/21 PR, 1/21 SD, 1/21 PD. 3-year EFS 49%; 3-year OS 60%	Despite impressive response rates, survival is not as good. Patients who do not achieve complete response with CT need additional therapies	(50)
	Japan 1997	1997-2006	28/0/28	2.79-15.1	Sx + CT + RT&CT + CT	5-year PFS 82.1%; 5-year OS 85.7%; 10-year PFS 78%; 10-year OS 82.1%;	Intensified CT including IT MTX concomitant with RT seems to allow a reduced CSI RT dose	(51)
	POG 9631	1998-2002	53/0/53	3-21	Sx + RT&CT + CT	5-year PFS 70.2%; 5-year OS 76.6%	This regimen is feasible and survival rates are similar to other regimens	(52)
	COG 99701	1998-2004	161/0/161	3.1-21.6	Sx + RT&CT + CT (VCR + CP)	Globally, 5-year PFS 77% for M1, 50% for M2 and 67% for M3; 5-year OS 83% for M1, 70% for M2 and 73% for M3 5-year PFS 71% (VCR+CP) 5-year OS 82% (VCR+CP) (P=0.36);	CBDCA as a radiosensitizer is a promising strategy. Non-difference between the two maintenance CT regimens	(53)
					Sx + RT&CT + CT (VCR + CP + CDDP)	5-year PFS 59% (VCR+CP+ CDDP); 5-year OS 68% (VCR+CP+CDDP) (P=0.36);		
	HART “The Milan Strategy”	1998-2007	33/0/33	3-34	Sx + CT + HFRT + CT	5-year PFS 72%; 5-year EFS 70%; 5-year OS 73%	HART combined with intensive CT is feasible and successful for metastatic medulloblastoma.	(54)
	CCG 99702	1999-2002	24/0/24	3-21	Sx + CT + RT&CT + CT	5-year EFS 46%; 5-year OS 50%	Prematurely closed because of high incidence of sinusoidal obstructive syndrome	(55)
	Korea	1999-2005	11/0/11	1.41-16.5	Sx + RT**^¶^** + CT	9/11 CR, 2/11 PR. 3-year EFS 83% (>3 years old); 3-year OS 83.3% (>3 years old)	High-dose CT may improve the survival of children with MB	(56)
	Egypt	2001-2004	48/0/48	Non specified	Sx + RT	3-year DFS 61.3% (RT only); 3-year OS 69.5% (RT only)	CT produced interruption of RT treatment due to myelosupression and, secondary, worse survival	(57)
					Sx + RT&CT	3-year DFS 48.9% (RT+CT); 3-year OS 48.4% (RT+CT)		
	Egypt	2001-2005	17/0/17	5-14	Sx + RT&CT + CT	3-year PFS 58.8%; 3-year OS 70.6%; 3-year PFS 63.6% for M0 and 50% for M+; 3-year OS 81.8% for M0 and 50% for M+	This approach is feasible, specially for M0 patients	(58)
	MET-HIT-2000-AB4	2001-2007	171/0/171	4.3-20.3	Sx + CT + HFRT&CT + CT	5-year EFS 62%; 5-year OS 74%	This scheme is feasible and conferred favorable overall survival	(59)
	French Gustave Roussy	2001-2010	21/0/21	4.3-20.4	Sx + CT + RT + CT**^¶^**	5-year EFS 65% and 5-year OS 74% (including 3 patients with PNET); For M+ MB 5-year EFS 72%; 5-year OS 83% (including 3 patients with PNET)	This scheme is feasible and successful	(60)
	HART UK	2002-2008	34/0/34	3-15	Sx + RT/(RT&CT**^¶^**) + CT	3-year EFS 59%; 3-year OS 71%	Could not reproduce the Italian results. EFS similar to other treatment strategies	(61)
	MGH-99-271	2003-2009	59/20/38	3-21	Sx + CT**^¶^** + RT/(RT&CT**^¶^**) + CT**^¶^**	5-year PFS 70%; 5-year OS 75%	Proton RT has similar outcomes and less toxicity	(62)
	Head Start III	2003-2009	92/24/66	0-10	Sx + CT + RT	5-year EFS 46%, 5-year OS 62%. 5-year EFS 61% in M0, 5-year OS 77% in M0; 5-year EFS 35% in M+, 5-year OS 52% in M+. 5-year EFS 50% in children < 6 years old; 5-year OS 65% in children < 6 years old; 5-year EFS 11% in children >= 6 years old; 5-year OS 36% in children >= 6 years old	Effective for young children without radiation. Worse survival rates for children > 6 years old	(63)
	SJMB03	2003-2013	330/227/103	3-21	Sx + RT + CT	5-year EFS 56.7%, 5-year PFS 58.7%, 5-year OS 69.5%. 5-year PFS WNT 100%, 5-year PFS SHH 25%, 5-year PFS group 3 40.6%, 5-year PFS group 4 68.1%	Molecular classification is essential in MB	(8)
	Korea	2005-2018	40/0/40	3-31.5	Sx + CT + RT + CT	5-year EFS 71.1%; 5-year OS 73.2%	Promising results in terms of low relapse/progression rate but high treatment-related mortality rate	(56, 64)
	India CBDCA	2005-2019	97/0/97	3-25	Sx + RT&CT + CT**^¶^**	5-year PFS 60.2%; 5-year OS 62.1%; 10-year PFS 46.3%; 10-year OS 48.8%. 5-year PFS 64.9% in M0/M1, 5-year OS 68.1% in M0/M1; 5-year PFS 53.4% in M2/M3, 5-year OS 53.3% in M2/M3.	CBDCA during RT is a simple and effective way of intensifying treatment	(65)
	Japan 2006	2006-2014	48/35/13	3-18	Sx + CT + RT&CT + CT**^¶^**	3-year PFS 100%; 3-year OS 100%	Intensified CT including intrathecal MTX concomitant with RT allows a reduced CSI RT dose	(66)
	ACNS0332	2007-2018	261/0/261	3-21	Sx + RT&CT (VCR) + CT	5-year EFS 66.4% (CBDCA), p=0.11. Globally, 5-year EFS 62.9%; 5-year OS 73.4%; 5-year EFS WNT 92.9%, 5-year EFS SHH 49.6%, 5-year EFS group 3 64.2%, 5-year EFS group 4 65.6%; 5-year OS WNT 100%, 5-year OS SHH 53.6%, 5-year OS group 3 73.7%, 5-year OS group 4 76.9%	CBDCA addition is beneficial only in group 3 MB CBDCA increases toxicity Molecular risk stratification is essential	(7)
					Sx + RT&CT (VCR+ CBDCA) + CT	5-year EFS 66.4% (CBDCA), p=0.11.		
	SIOP PNET HR+5	2009- 2021	51/0/51	5-20	Sx + CT + RT + CT	5-year PFS 76%; 5-year OS 76%; 5-year PFS WNT 100%, 5-year PFS SHH aprox 72%, 5-year PFS group 3 aprox 54%, 5-year PFS group 4 100%	This approach with high-dose CT + conventional RT obtains high survival rates	(67)
	SJMB12 stratum W3, N3 or S2	2013- present	No results yet	3-39	Sx + RT + CT	No results yet	Results pending	–

AR, average-risk; CBDCA, carboplatin; CCNU, lomustine; CDDP, cisplatin; CP, cyclophosphamide; CR, complete response; CSI, craniospinal irradiation; CT, chemotherapy; DFS, disease-free survival; EFS, event-free survival; HR, high-risk; IT, intrathecal; MB, medulloblastoma; MTX, methotrexate; N, number of patients included; NR, non response; OS, overall survival; PD, progressive disease; PF, posterior fossa; PFS, progression-free survival; PNET, primitive neuroectodermal tumors; PR, partial response; RT, radiation therapy; RT&CT, radiation therapy with concomitant chemotherapy; SD, stable disease; Sx, surgery; VCP, vincristine + lomustine + prednisone; VCR, vincristine; ^¶^, only in some patients.

**Figure 2:**
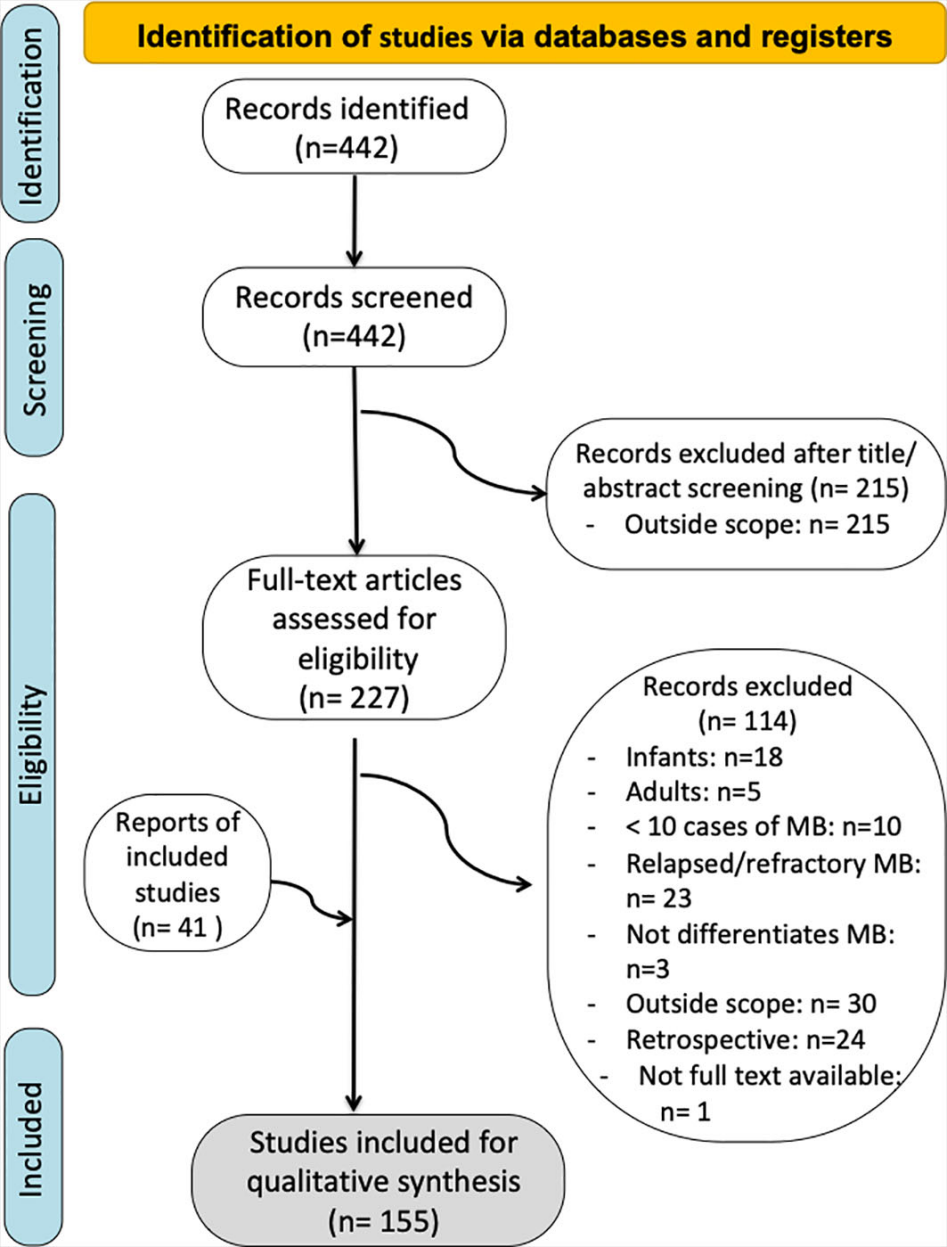
Preferred reporting items for systematic reviews and meta-analyses (PRISMA) flow diagram.

Around 1960, the standard of care of MB consisted on surgery and post-operative craniospinal irradiation (CSI), with a 5-year overall survival (OS) being around 30-50% (16, 20–22). In 1975, the first multi-center randomized trial of the International Society of Pediatric Oncology (SIOP I (16)) was designed. Since then, survival rates are better (**Figure 2**).

The aim of this paper is to review, synthesize and analyze the design and outcome of the different treatment protocols used in 0-18 year-old children affected with MB. Therefore, we would critically understand the rationale for the current management of children with MB. In this regard, we conducted a systematic review, analyzing the clinical trials and multicenter studies performed since the 1940s, their design, hypothesis and conclusions. Since treatment is considerably different in children under 3 years of age, for the purpose of this review we will exclude trials based exclusively in infants and children younger than 3 years of age.

## 2 Materials and methods

### 2.1 Search strategy

**Figure 3:**
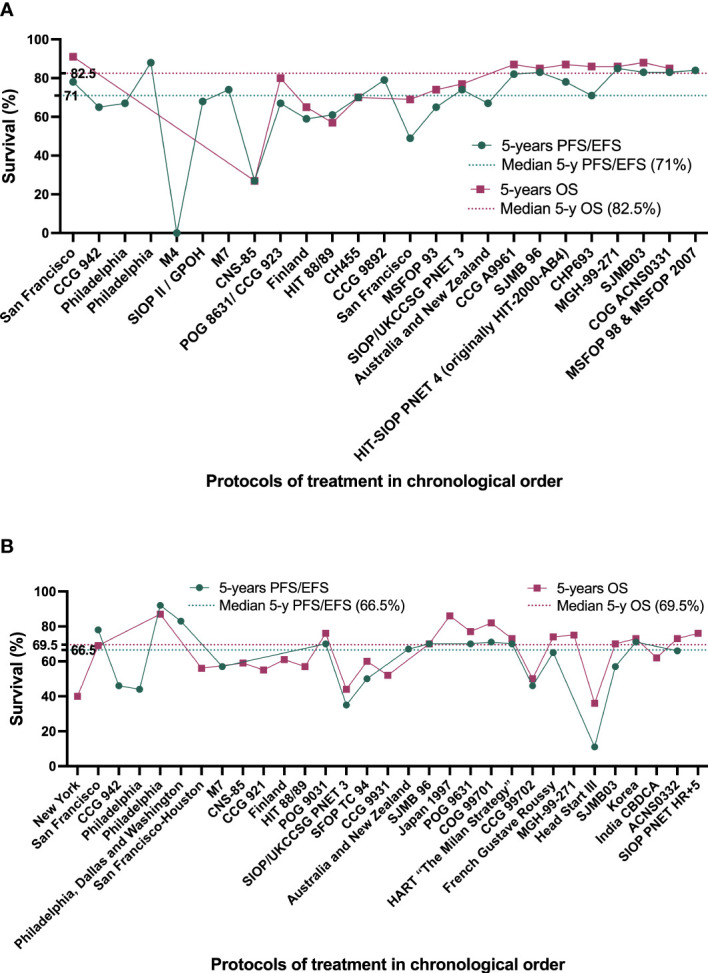
Best survival rates achieved by the different treatment protocols developed since 1963 by chronological order. **(A)** treatment protocols for patients with average-risk MB (AR-MB). **(B)** treatment protocols for patients with high-risk MB (HR-MB).

We performed a comprehensive search through the indexed database PubMed Central in accordance with the Preferred Reporting Items for Systematic Reviews and Meta-Analyses (PRISMA) statement guidelines (68) (**Figure 3**). The research string included the word “medulloblastoma”. Afterwards, we selected the following filters: “clinical trial”, “randomized controlled trial” and “multicenter study”, so that all clinical trials and prospective studies were included. We initially conducted the search on January 31^st^, 2022 and we updated it on May 11^th^, 2023.

### 2.2 Study selection

Two investigators (MPOM and LIML) read the titles and abstracts of all the papers that met the criteria. We selected articles written in any language focusing on outcomes and/or side effects after a defined treatment, randomized or not. There were no restrictions regarding the date of publication, language or country of origin of the articles. We subsequently excluded studies focusing exclusively on supratentorial PNETs, studies on refractory/relapsed tumors, studies focused exclusively on patients younger than 3 years of age, studies focused exclusively on patients older than 18, studies involving different tumors without differentiating survival according to different histologies, studies including fewer than 10 cases of MB, trials on different types of PNETs, studies based solely on retrospective data and those without reference to outcome and/or side effects after a defined treatment.

### 2.3 Data extraction and quality assessment

The selected papers were deeply reviewed by MPOM. Their bibliographies were also manually screened for other relevant studies to ensure that the search was complete. We extracted the following information from each clinical trial: trial name, dates the trial was open, number of patients included, patient risk classification, doses of RT administered (craniospinal, boost, fractions, boost area and duration), type of CT administered (before RT, after RT and/or concomitant to RT, doses and timing of each drug), and main conclusions of the trial. When available, the rationale of each treatment protocol was also analyzed.

## 3 Results

The total selection process is summarized in **Figure 2**. A total of 442 papers were included for initial review. Title and abstract screening resulted in exclusion of 215 studies. Therefore, 227 articles concerning the outcomes and conclusions of the different treatment protocols over time were selected for full-text review. Subsequently, 113 articles were excluded for meeting one or more exclusion criteria. One article was not available in full-text version. A supplementary search of the reference list generated 41 additional papers. Finally, a total of 155 papers were included to develop this systematic review. Based on the information gathered, we answer some of the key questions regarding MB and its management over time.

## 4 Discussion

### 4.1 What are the main poor-prognosis factors in medulloblastoma?

One of the milestones in the management of MB has been the classification of patients into prognostic groups, in order to obtain the best survival rates without unnecessary long-term toxicity. Currently, the five main poor-prognostic factors are: age less than 36 months, metastatic disease (according to Chang Stage), postoperative residual tumor volume ≥1.5 cm^2^, anaplastic histology and MYC amplification (1). The San Francisco (9) treatment protocol (1966-1987), the European SIOP I (16) trial (1975-1979), and the American CCG 942 (21) trial (1975-1981) were the first trials that defined some of these poor prognosis factors (9, 16, 21) (**Tables 1**, **3**, **4**).

The first studies that adjusted treatment based on the risk were performed at the Children’s Hospital of Philadelphia (22) (1983-1991), the Children’s National Medical Center in Washington (24) (1988-1993) and the Children’s Medical Center in Dallas (24) (1988-1993). In these studies, children were classified into “standard-risk” or “poor-risk” groups (22, 24). Children older than 5 years of age in the standard-risk group were treated with RT alone, whereas children younger than 5 years of age in the standard-risk and children older than 18 months in the poor-risk group were treated with RT plus CT. There was a major difference in outcomes between children treated with RT alone and those treated with RT+CT: 5-year disease-free survival (DFS) was 52% and 88%, respectively (22) (**Tables 3**, **4**). This is even more remarkable considering that 82% of patients of the latter group met the poor-risk criteria (22). Since then, patients have been classified into average/standard risk (AR-MB) and high-risk (HR-MB) groups. Lately, given the excellent prognosis of certain molecular subtypes, some protocols have created a new low-risk group (LR-MB) (5, 85).

Disease extent was shown to be important both in the Philadelphia-Washington-Dallas (22, 24) treatment plan (5-year progression-free survival (PFS) of 90% for patients with localized disease Vs. 67% in metastatic disease (24)) and in the CCG 921 (32) randomized phase III study (5-year PFS for M0, M1 and M2 was 70%, 57% and 40%, respectively (32)). This has subsequently been confirmed in posterior trials, such as the French Society of Pediatric Oncology (SFOP) study of 1993 (45) which confirmed that M1 tumors should be considered high-risk (**Tables 3**, **4**).

Another prognostic factor is the postoperative bulky residual tumor size (≥1.5 cm2) in M0 tumors: 5-year PFS were 78% and 54% depending on whether the residual tumor was less than 1.5 cm2 or not in the CCG 921 (32) study. Similarly, the 5-year PFS of patients who achieved a complete response with CT before RT was 57% in the HIT 88/89 trial, compared with 20% in patients that did not achieve it (34). In contrast, the presence of residual disease in patients M0 was not associated with an inferior outcome in the POG 9031 (40) trial. Although this has traditionally been stated, there are some recent reports evidencing that the residual tumor size may be of less importance than initially thought (8, 86), especially after taking into account molecular subgroup classification (87) (**Tables 3**, **4**).

The presence of anaplasia is also considered a high-risk feature, even in patients who otherwise fulfill standard risk criteria (8, 53, 77).

Children younger than 36 months old have worse prognosis than older children (3). The biology of MB in infants is different than that of older children and they present more frequently with metastatic disease. Moreover, they mostly receive radiation-sparing treatment protocols based on CT and high-dose CT due to the high susceptibility of RT-induced neurocognitive deficits in these young children (3, 4). For all this, age under than 36 months old is considered to be one of the main poor prognostic factors. Many reports outline the behavioral and prognostic differences between infants, children and adults (88).

Prolonged duration of symptoms before diagnosis does not seem to be a poor prognostic factor. On the contrary, it has been associated with lower metastatic stages, some specific molecular subgroups and better survival rates (89, 90).

### 4.2 Is medulloblastoma always the same disease?

An extraordinary milestone in the history of MB has been the definition of its molecular subtypes (5). This recent molecular classification enables us a deeper understanding of tumor biology and pathophysiology. What was originally thought to be a single disease is now up to 12 different diseases (6). This classification is going to become increasingly important over time. This has recently been demonstrated by the results of the SJMB03 (8) (2003-2013) and the ACNS0332 (7) (2007-2018) trials. These trials document that survival rates differ dramatically between molecular groups. In this regard, the ACNS0332 (7) trial found 5-year event-free survival (EFS) rates of 92.9% for WNT, 49.6% for SHH, 64.2% for group 3 and 65.4% for group 4 (p=0.06). Moreover, it has been observed that prognosis can be excellent in patients with MB of a specific molecular subgroup, despite presenting high-risk features. In this respect, the SJMB03 trial found a 5-year PFS in high-risk WNT-MB of 100%, compared to only 67% in standard-risk group 3-MB (8). The high-risk group of patients treated with the SJMB03 trial show dramatic differences in survival, with 5-year PFS rates of 100% for WNT and <30% for SHH (8) (**Tables 3**, **4**).

The excellent survival rates observed in the WNT molecular subgroup has led to the definition of a new low-risk treatment group. This low-risk group includes patients with molecular WNT-MB (defined by the presence of nuclear beta-catenin positivity by immunohistochemistry and the CTNNB1 mutation, and, in some protocols, associated with the presence of monosomy 6) without any high-risk features (i.e. age >= 16 years old, large-cell-anaplastic histology, metastatic disease, MYC amplification and/or residual tumor volume >1,5 cm2). There are currently 3 ongoing trials based on a de-escalated treatment for this good-prognosis WNT subgroup: SJMB12 stratum W1 (NCT01878617, from 2013 to present), PNET5 MB-LR (NCT02066220, from 2014 to present) and COG ACNS1422 (NCT02724579, from 2017 to present) trials (**Table 2**). Molecular subgrouping is the future and should be taken into account when defining treatment intensity.

### 4.3 Is medulloblastoma a curable disease?

**Figure 4:**
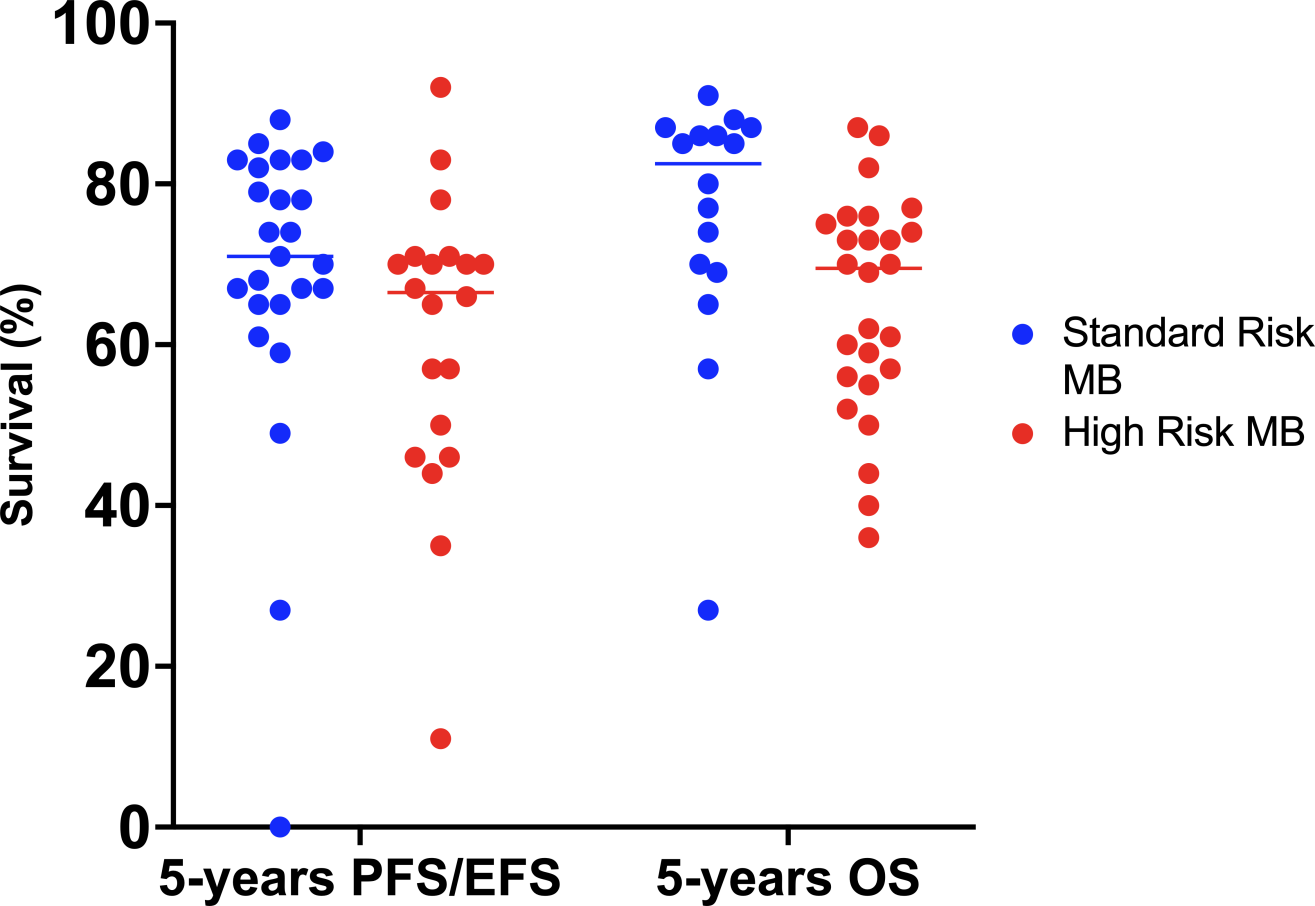
Best survival rates achieved in the different treatment protocols since 1970. EFS, event-free survival; MB, medulloblastoma; OS, overall survival; PFS, progression-free survival.

Survival rates have improved greatly over time. Overall, in children older than 3 years of age, 5-year OS is around 70-85% in AR-MB and 60-65% in HR-MB (1, 2, 91) (**Figure 4**). Approximately two-thirds of patients with MB fall into the standard-risk group.

Considering molecular subtype, patients with AR-MB have a 5-year PFS of 100% for WNT-MB, 77.5% for SHH-MB, 66.7% for group 3 MB and 87.3% for group 4 MB. For patients with HR-MB, the 5-year PFS rates are 100% for WNT-MB, 25% for SHH-MB, 40.6% for group 3 MB and 68.1% for group 4 MB (8).

Recurrences occur in about 30% of children and can occur >5 years after diagnosis. There is usually only one chance to cure children with MB; survival is poor in case of relapse (<10% al 5 years, even for initial standard-risk patients) (2, 7, 91).

### 4.4 Do we need and experienced neurosurgeon or anyone is good enough?

The importance of good surgery was soon demonstrated. The University Hospitals of Philadelphia and Pennsylvania incorporated in December 1974 the use of computer tomographic scan for the planning of the tumor surgical approach and the use of binocular operating microscope. These two techniques dramatically improved patients survival (4-year PFS of 84% with these techniques Vs. 38% without) (15). The use of novel technologies, such as navigated intraoperative ultrasonography, should be implemented, as they improve the complete response rate and lower the morbidity (92).

Similarly, the SIOP I (16) trial (1975-1979) found statistically significant differences in survival depending on where the children were treated (5-year OS in major centers 57% Vs. 42% in minor centers) (16).

### 4.5 Is radiation therapy the main cornerstone of treatment? How and when should be administered?

**Figure 5:**
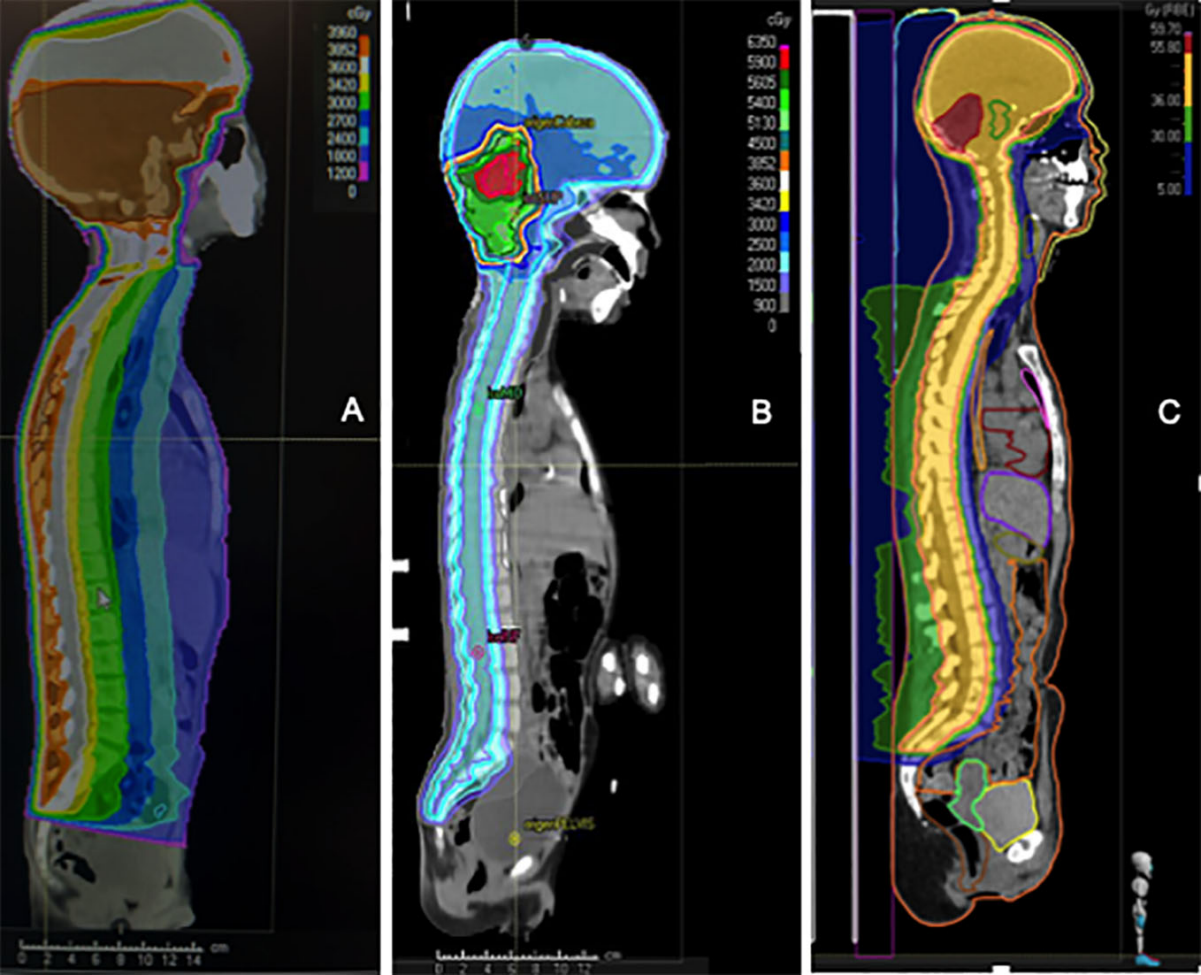
Comparison of the dosimetry of three patients with medulloblastoma who received craniospinal irradiation with a local boost. **(A)** old-fashioned 3D Conformal Radiation Therapy (3D-CRT) with a unique posterior photon beam. **(B)** modern Intensity-modulated radiation therapy (IMRT) photon technique; **(C)** modern proton technique.

The introduction of post-operative craniospinal irradiation (CSI) in the management of MB started to give the patient some chance of cure (20). The Head Start III trial (2003-2009) obtained very poor survival rates in children between 6-10 years old (5-year EFS 11%, 5-year OS 36%) (63). This was probably secondary to the absence of RT (it was only indicated in patients ≥6 years old with residual disease) or its very late administration (after 5 cycles of CT and one cycle of high-dose CT with stem cell rescue). Therefore, it can be stated that RT in MB is an essential part of the treatment. It is included in the treatment plan of every child older than 3 years of age affected by MB. The RT technique, dosing schedule, boost extension and even patient positioning (93) have changed over the time. The RT technique, dosing schedule, boost extension and even patient positioning (93) have changed over the time (**Figure 5**).

Craniospinal RT is delivered since the 1940s, in accordance with post-mortem findings that revealed tumor spread when the entire brain and cord were not treated (10). RT should include some high-risk areas for recurrence, such as cribriform plaques or temporal lobes (91).

RT in MB should be administered early and without interruptions. The timing of RT should not be delayed by any cause, not even by the administration of adjuvant CT because otherwise survival decreases. This was demonstrated in the HIT-SIOP PNET 4 and HIT 91 trials (42, 43, 81) (**Tables 3**, **4**). The current recommendation is to start RT no later than 40 days after surgery (ideally within 28 days of surgery) (91). Similarly, the SIOP/UKCCSG PNET3 study also demonstrated that prolonging the duration of RT more than 50 days had a negative impact on outcome and should be avoided (94) (**Tables 3**, **4**).

RT should be administered in experienced centers and with strict quality control, in order to avoid relapses secondary to radiotherapeutic errors, as experienced in the past (95). One study found that 23% of patients received less than the 95% of the prescribed dose (96). Targeting deviations occur in 49-71% of patients, so it is essential to use quality control techniques (97, 98). The quality of RT technique is strongly correlated with outcome (97).

### 4.6 Which dose of radiation therapy is optimal?

Early protocols showed improved survival rates with increasing the RT dose to the posterior fossa and spinal axis (12, 20). Subsequently, and because of the deleterious effects of RT on the developing brain, various groups have attempted to reduce the RT dose to reduce long-term sequelae. Nowadays, craniospinal RT doses depend on prognostic groups. Doses of 18 Gy, 23.4 Gy and 36 Gy, at 1.8 Gy/fraction, are generally considered adequate for low-risk, standard-risk and high-risk patients, respectively, with boost in the primary tumor up to 54-55.8 Gy (1, 2).

**Figure 6:**
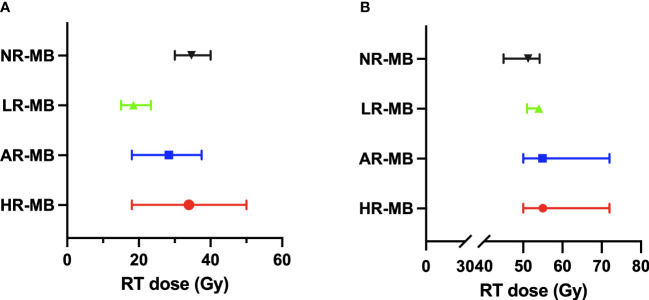
Radiation Therapy (RT) doses administered in the different treatment protocols classified by risk. The median dose is shown with the range between the maximum and minimum dose. **(A)** Craniospinal RT dose. **(B)** Boost RT dose. AR-MB, average-risk medulloblastoma; Gy, gray; HR-MB, high-risk medulloblastoma; LR-MB, low-risk medulloblastoma; NR-MB, non-defined risk medulloblastoma.

Initially, the craniospinal RT dose was 35-36 Gy. Since then, various clinical trials have been developed reducing the dose of CSI (**Figure 6**). One of the first studies reducing craniospinal RT dose was performed in San Francisco (9) (1966-1987) and found no increased tumor recurrence rate despite reducing CSI from 30-40 Gy to 25 Gy combined with CT both in standard and high-risk patients (9). Although not reaching statistical significance between both groups as a whole, DFS rates in the high-risk group did differ (5-year DFS of 78% for standard CSI dose Vs. 39% in reduced-CSI dose and CT) (9) (**Tables 3**, **4**).

In patients with AR-MB, there are several treatment protocols that randomized different doses of RT (**Figure 6**). The POG 8631/CCG 923 (70, 71) trial (1986-1990) attempted to reduce the craniospinal RT dose from 36 to 23.4 Gy (without any CT treatment), but it was prematurely closed due to a significant increase in the risk of early relapse in the reduced-dose RT group detected in the interim analysis in 1990 (73). Posterior analysis revealed that over time these differences narrowed, reaching no statistical difference in 8-year EFS (67% for 36 Gy and 52% for 23.4 Gy) (71). The SIOP II (26) trial (1984-1989) also randomized the craniospinal RT dose (35 Vs. 25 Gy) and demonstrated a non-statistically significant better outcome in those with standard craniospinal RT dose (5-year EFS was 67.6% Vs. 55.3%) and a particularly poor outcome in patients who received both pre-irradiation CT and low craniospinal RT dose (5-year EFS 41.7%) (26). Subsequently, the CCG 9892 (73) pilot study (1989-1994) found similar survival rates when a reduced dose of craniospinal RT (23.4 Gy) was combined with concurrent VCR and followed by maintenance CT based on the combination of CDDP, VCR and CCNU (73). This was later confirmed in the phase III CCG A9961 (77, 78) study (1996-2000). In Europe, the MSFOP 93 (74) study (1991-1998) reduced the craniospinal RT dose to 25 Gy, preceded by CT, with worse results (5-year PFS of 64.8%) (74). Concurrently, the multicenter SJMB 96 (49, 79) trial (1996-2003) combined reduced-dose craniospinal RT with dose-intensive CT, achieving a 5-year EFS rate of 83% (49, 79) (**Tables 3**, **4**).

Subsequent attempts to further reduce the CSI dose to 18 Gy have shown an increased risk of relapse, according to the results observed in the CHP455 (72) pilot trial (1988-1990), in the single-arm pilot study CHP693 (82) (2001-2010), and in the COG ACNS0331 (83) randomized phase III trial (2004-2012) (**Table 4**). Interestingly, children in the CHP455 pilot trial who did not relapse in the first two years, did not relapse in the following ten years and show less neurocognitive impairment.

These different trials demonstrate that it is feasible to use slightly reduced doses of craniospinal RT (23.4 Gy) in standard-risk patients if CT is added after RT, with or without concomitant CT to RT. This approach has become the standard of care for patients with standard-risk MB (1, 2, 99).

In patients with HR-MB, craniospinal RT doses are higher (**Figure 6**). The most frequent CSI dose is 36 Gy (range 35–39 Gy). In the San Francisco-Houston (27) phase II study (1984-1992), the reduction of CSI dose to 24 Gy resulted in decreased survival (27). Conversely, the Japanese Pediatric Brain Tumor Consortium (51, 66) (1997-2006 and 2006-2014) despite administering a reduced neuraxis dose of 18 Gy, obtained optimal survival rates [5-year PFS of 82.1% (51)] (**Table 4**).

In the Philadelphia-Washington-Dallas (Packer et al., 1991, 1994) treatment plan (1983-1989 in Philadelphia and 1988-1993 in Washington-Dallas), RT doses differed according to patient age (full-dose was 36 Gy CSI with a local boost up to 54-55.8 Gy and reduced-dose was 23.4 Gy and 50.4-55.8 Gy of boost), with no differences observed in outcome (Packer et al., 1994) (**Table 4**).

Currently, the standard of care for high-risk patients remains 36 Gy of craniospinal RT dose plus a boost to the primary tumor and the bulky metastases (2).

In patients fulfilling low-risk features, the RT dose has been further reduced (to 15 Gy craniospinal with a 51 Gy boost to the TB in the SJMB12 trial stratum W1; and to 18 Gy craniospinal with a 54 Gy boost to the TB in the ACNS1422 and PNET5 MB-LR (**Table 2**). The results of these trials are still pending.

Attempts to further reduce the craniospinal RT dose should be undertaken with caution, to avoid repeating the negative experiences of the past (25, 70–72, 83).

### 4.7 Is it safe to reduce the boost area to the tumor bed?

**Figure 7:**
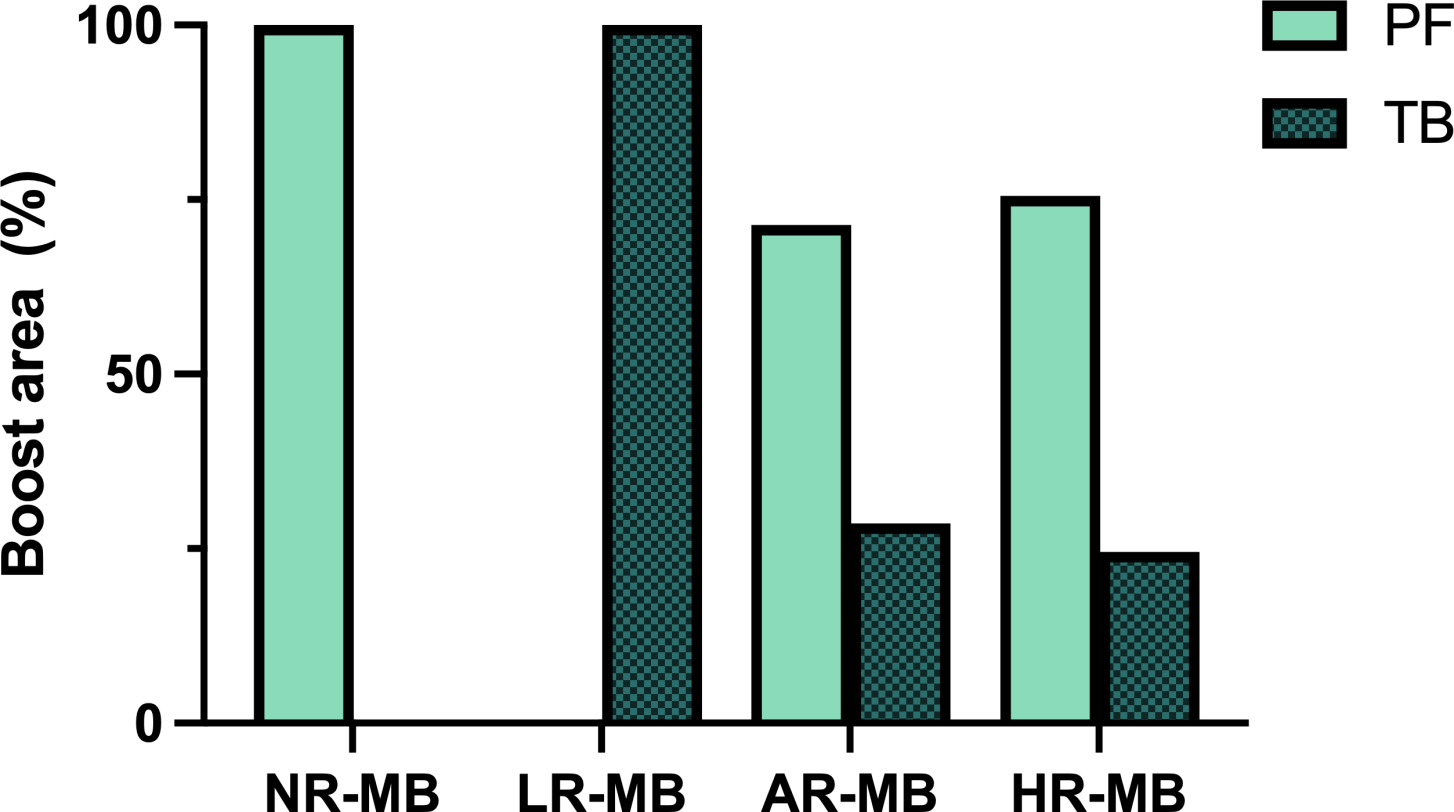
Boost area administered in the different treatment protocols. AR-MB, average-risk medulloblastoma; HR-MB, high-risk medulloblastoma; LR-MB, low-risk medulloblastoma; NR-MB, non-defined risk medulloblastoma; PF, posterior fossa; TB, tumor bed.

RT administered exclusively to the tumor bed (TB) plus a safety margin rather than to the entire posterior fossa (PF) has been administered in several studies (**Figure 7**) and is today the standard of care (83). The goal of this reduction is to decrease toxicity without compromising outcome. This safe reduction had been debated since the late 1990s (1, 2, 91, 100, 101). The non-randomized trials SJMB 96 (49, 79) and MSFOP 98 (80) had had positive results (5-year cumulative incidence of PF failure of 4.9% (79) and 0% (80), respectively). Finally, its safety was demonstrated in the randomized phase III COG ACNS0331 (83) trial: the 5-year EFS for involved field RT and PF irradiation was 82.5% and 80.5%, respectively (83). However, according to the results of the French M4 (25) protocol (1984-1985), RT should always include the supratentorial area because otherwise the outcome is very poor (6-year DFS of 18% (25)) with a high incidence of relapse within the supratentorial area (**Tables 3**, **4**). Nevertheless, RT administered exclusively to the tumor site is not sufficient, and CSI should always be performed.

### 4.8 Does hyperfractionated radiation therapy have a role?

Subsequently, with the improvement in RT techniques, some groups began to evaluate the effect of hyperfractionated RT (HFRT). Some of the first studies include the New York pilot study (36) (1989-1995) and the San Francisco phase II study (38) (1990-1992). The New York trial included patients with high-risk features and consisted of HFRT followed by CT. They found excellent long-term PFS in the subgroup of patients with high T stages but M0 (6.5-year PFS of 95%) (36). The San Francisco trial included patients with AR-MB and HR-MB and consisted of HFRT that was only followed by maintenance CT in the poor-risk group. They observed lower PFS rates in the AR-MB group and no superior outcomes in the HR-MB group compared to other studies (38). Based on the results of the two aforementioned pilot trials (36, 38), the CCG 9931 (46) phase II pilot study (1994-1997) for patients with HR-MB combined 5 cycles of pre-RT CT with HFRT over 2 months and achieved a 5-year EFS of 43% (46) (**Tables 3**, **4**).

In AR-MB, the French started using HFRT in 1998 in the non-randomized MSFOP 98 (80) treatment protocol (1998-2001) and observed very good 6-year EFS rates (75%) despite avoiding CT (80). Additionally, the randomized multicenter HIT-SIOP PNET 4 (81) trial (2001-2006) compared the traditional RT schedule (CSI 23.4 Gy and up to 54 Gy in posterior fossa) with the hyperfractionated schedule (1 Gy twice a day over 48 days) plus concurrent weekly VCR and maintenance CT in both treatment arms. In this study, HFRT was not superior to conventional RT [5-year EFS was 78% for HFRT and 77% for conventional RT (81)] (**Table 3**). Therefore, conventional RT remained the standard of care.

Further on, the efficacy and feasibility of Hyperfractionated Accelerated RT (HART) for metastatic MB was tested. It was first used by the Italians (1998-2007 (54), who combined it with pre-RT CT and maintenance CT with good tolerance and promising results [5-year EFS of 70% (54)]. Subsequently, the British (61) (2002-2008) used the same strategy of RT followed by maintenance CT but were unable to reproduce the Italian excellent results [3-year EFS of 59% (61, 102)] (**Table 4**).

Overall, neither HFRT nor HART have shown to be more beneficial than the standard administration scheme, so they are not currently recommended (81).

### 4.9 Is proton therapy superior to photon therapy?

Technological advances have led to the development of proton RT, which reduces the damage to surrounding healthy tissue. Disease control and outcomes are similar with proton and photon RT in AR-MB (103). Proton RT has been used in MB in the non-randomized phase II trial MGH-99-271 (62) (2003-2009), with similar survival rates (5-year EFS of 85% patients with AR-MB and of 70% for patients with high-to-intermediate risk MB) and lower toxicity rates for the entire cohort. They report less grade 3-4 hearing loss (16% at 5 years Vs. 24% in the CCG A9961), superior intellectual outcome and no proton-induced cardiac, pulmonary or gastrointestinal toxicity (62). However, these results are from patients with different ages, RT doses, boost volumes and follow-up time, so the results should be interpreted with caution. Furthermore, the comparison is against outdated photon RT techniques. The comparison between proton RT and advanced photon techniques (such as Intensity Modulated Radiation Therapy [IMRT] or Volumetric Modulated Arc Therapy [VMAT]) has not been explored. In any case, proton RT is effective but not without side effects; neuroendocrine deficits were still reported in 55% of patients at 5-year follow-up (62). Both photon and proton RT are effective in the treatment of MB.

### 4.10 Does chemotherapy have a role in the management of medulloblastoma?

Precisely, this is the first question that international groups tried to answer. The Royal Marsden Hospital (11) developed a pilot study with 37 patients to test the feasibility of administering concurrent and adjuvant CT. This pilot study achieved a very significant improvement in 5-year OS (71%) (11) compared to historical control (32%) and was the basis for the SIOP I trial (**Table 1**).

In 1975, two parallel prospective randomized trials were developed with the aim of confirming that CT improved survival: the European SIOP I (16) trial (1975-1979), and the American CCG 942 (21) trial (1975-1981). In both studies, there were two randomized arms: surgery and RT Vs. surgery, RT with concurrent VCR and followed by 8 cycles of adjuvant CT every 6 weeks based on CCNU and VCR and, in the American, associated with prednisone (**Tables 3**, **4**) (16, 21). The SIOP I trial was prematurely closed in 1979 because there was a significant difference in the event-free survival (EFS) of both arms (53% for the arm with CT and 48% for the arm without CT, p=0.005). Subsequent follow-up revealed relapses in the CT group, narrowing the difference in EFS between both arms. Nevertheless, CT was proven to be beneficial in some specific subgroups (16). The CCG 942 trial concluded that CT was only beneficial for metastatic MB (21). Similarly, the randomized POG 7909 (17) trial (1979-1986) also found a benefit towards CT, in this case based on the MOPP regimen (nitrogen mustard, VCR, prednisone and procarbazine) (17). The Philadelphia-Washington-Dallas (22, 24) treatment plan (1983-1989 in Philadelphia and 1988-1993 in Washington-Dallas) combined CT and RT for children classified into the “poor-risk” group (22). They received RT with concomitant VCR and 8 cycles of adjuvant CT every 6 weeks based on CCNU, CDDP and VCR. In this study, patients with metastatic MB achieved a 5-year PFS of 67% (24) (**Tables 1**, **3**, **4**).

**Figure 8:**
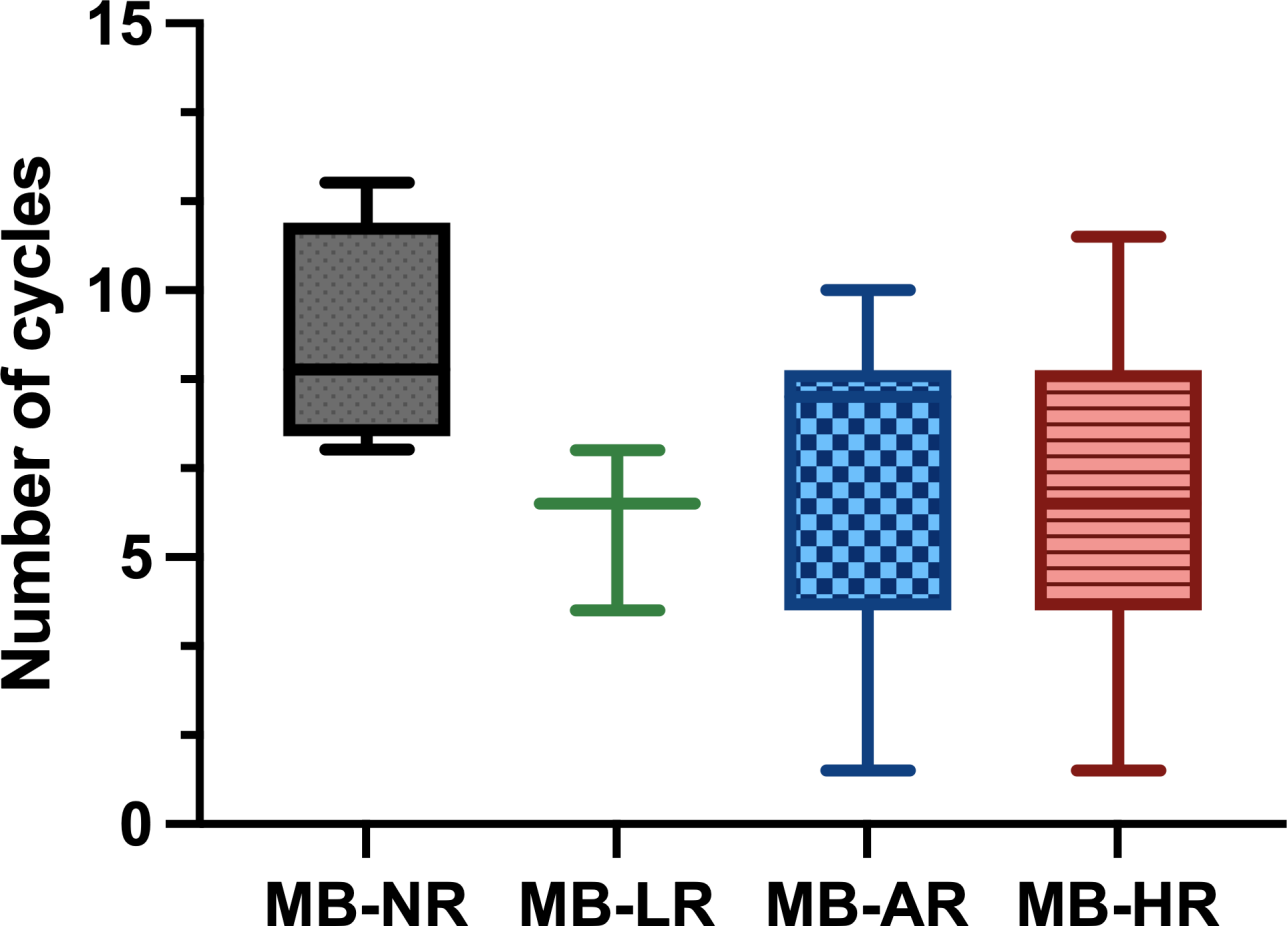
Number of chemotherapy (CT) cycles administered in the different treatment protocols classified by risk. The median dose is shown with the range between the maximum and minimum dose. Gy, gray; MB-AR, average-risk medulloblastoma; MB-HR, high-risk medulloblastoma; MB-LR, low-risk medulloblastoma; MB-NR, non-defined risk medulloblastoma.

Based on the multiple studies outlined, we can conclude that both CT and RT are effective in MB and that the combination of both has a synergistic effect. CT has been shown to be beneficial in the treatment of MB, especially in the high-risk subgroup (16, 21). The number of cycles administered varies in the different treatment protocols (**Figure 8**).

### 4.11 Which chemotherapy schemes are the most appropriate?

**Figure 9:**
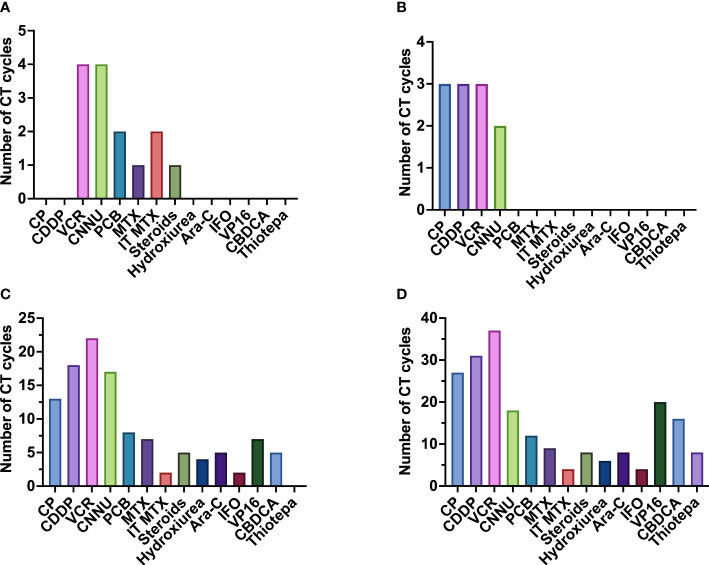
Frequency of administration of the different chemotherapeutic drugs administered, divided by risk. **(A)** chemotherapeutic drugs used in non-defined risk medulloblastoma (NR-MB); **(B)** chemotherapeutic drugs used in low-risk medulloblastoma (LR-MB); **(C)** chemotherapeutic drugs used in average-risk medulloblastoma (AR-MB); **(D)** chemotherapeutic drugs used in high-risk medulloblastoma (HR-MB). Gy, gray; Ara-C, cytarabine; CBDCA, carboplatin; CCNU, lomustine; CDDP, cisplatin; CP, cyclophosphamide; IFO, ifosfamide; IT-MTX, intrathecal methotrexate; MTX, methotrexate; PCB, procarbazine; VCR, vincristine; VP-16, etoposide.

Suitable CT regimens are based on the combination of multiple drugs, the most commonly used being vincristine (VCR), cisplatin (CDDP) and cyclophosphamide (CP) (**Figure 9**). Although each treatment protocol has its peculiarities, administration of 6 to 9 cycles of a combination of CDDP, CCNU, VCR or CP every 4-8 weeks after completion of RT is a frequent approach (SIOP I (16), CCG 942 (21), Philadelphia-Dallas-Washington (24), San Francisco ‘90 (38), CCG 9892 (73), New York ‘89 (36), HIT 91 (42, 43), CCG A9961 (77, 78), HIT SIOP PNET4 (81), MET-HIT-2000-AB4 (59), COG 99701 (53), COG ACNS0332 (7), COG ACNS0331 (83), SJMB12, PNET5 and COG ANCS1422) (**Tables 1**–**4**).

Carboplatin (CBDCA) has been used instead of CDDP in an attempt to diminish CDDP-derived ototoxicity and nephrotoxicity. The Italian MB group tested it a long time ago (1988-1992) with good results in some patients (1 complete response, 7 partial responses, 4 stable disease and 1 progressive disease after 2 cycles) (35). Afterwards, two other studies tested the use of CBDCA: the investigational protocol in St. Jude (39) (which administered CBDCA prior to RT in patients with HR-MB) and the American study (76) (which administered CBDCA after RT in patients with AR-MB). Both studies obtained disappointing results. The American Study failed to achieve better results than with CDDP (76). In the St Jude protocol, they observed a slightly increased risk of progressive disease, although results should be interpreted with caution due to the reduced number of patients included (39).

Some CT regimens have been tested in the subgroup of high-risk patients with the aim of increasing survival by rising intensity. One of these regimens includes the intensive “8-in-1” regimen, that includes CP, CDDP, VCR, CCNU, procarbazine, steroids, hydroxyurea and cytarabine. This regimen has been part of different studies (SIOP II (26), M7 (28, 29), CCG 921 (32), SFOP (45), among others). The CCG 921 compared the “8-in-1” regimen (administered before and after RT) with the VCP regimen after RT (based on VCR, CCNU and prednisone). The VCP regimen was superior to the “8-in-1” scheme [5-year PFS was 63% and 45%, respectively (32)] (**Table 4**).

However, the studies have mostly been single-arm clinical trials, so it is not possible to fully compare different CT schemes. When randomized studies comparing two different CT schemes have been performed, the results have either shown non-statistical difference between the two approaches (e.g., CCG A9961 (78), COG 99701 (53)) or the differences have been mainly due to different timing in RT administration (e.g. HIT 91 (42, 43), CCG 921 (32)) (**Tables 3**, **4**).

### 4.12 How and when should the chemotherapy be administered?

**Figure 10:**
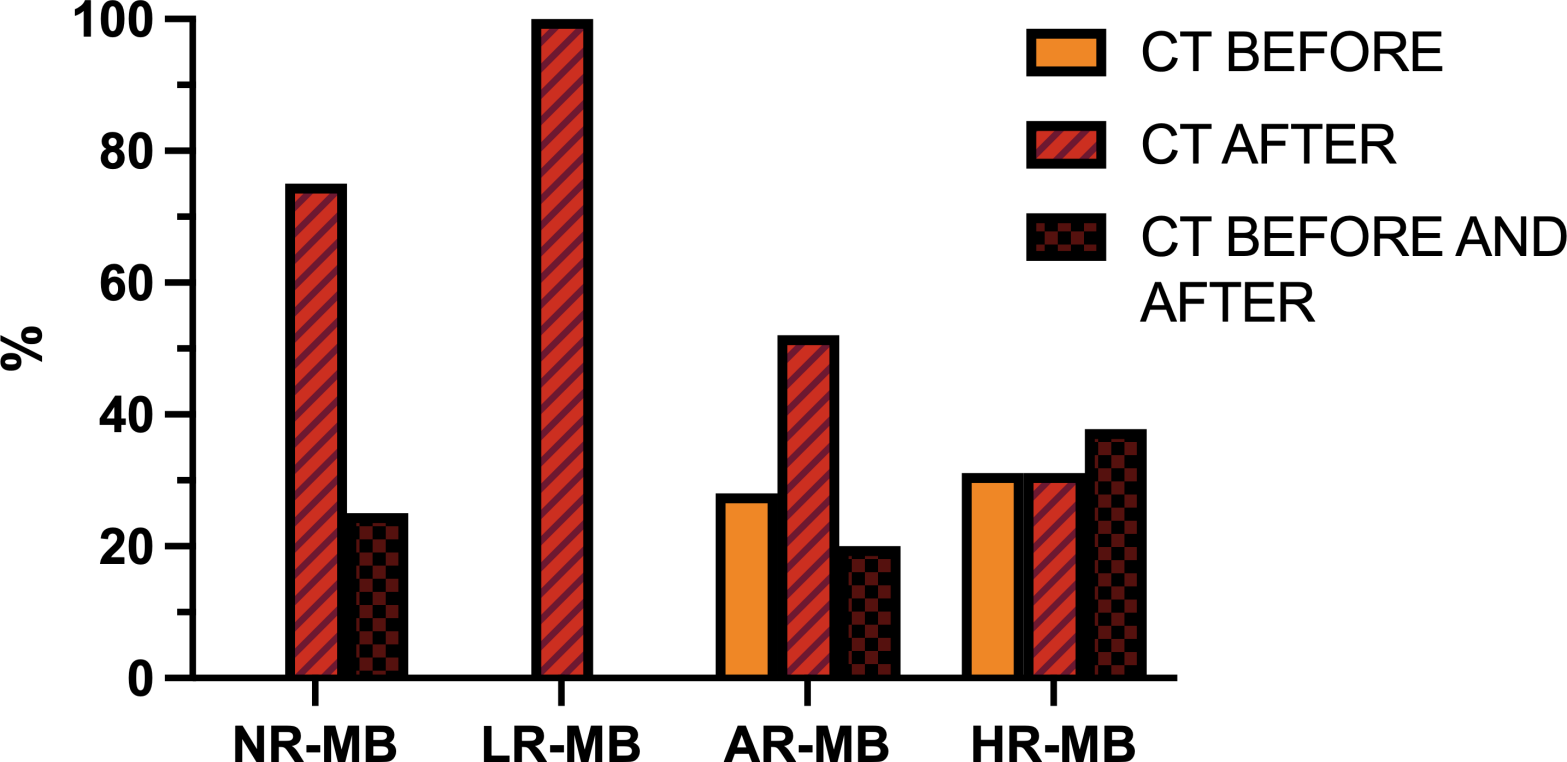
Timing of chemotherapy (CT) with respect to radiation therapy administration (before, after or both), divided by risk. AR-MB, average-risk medulloblastoma; HR-MB, high-risk medulloblastoma; LR-MB, low-risk medulloblastoma; NR-MB, non-defined risk medulloblastoma.

Over time, there have been several attempts to assess whether pre-irradiation CT (“sandwich CT”) improved survival (**Figure 10**). The first studies to test this were the GPO-MBL 80 (18, 19) study (1980-1983) and the randomized multicenter SIOP II (26) trial (1984-1989). In the German study, all patients received pre-RT CT based on procarbazine, VCR and methotrexate (MTX), but were randomized to receive or not CT after RT. In the SIOP II, the standard-risk group were randomized to receive RT alone versus sandwich CT based on the same drugs used in the German study, while children in the high-risk group were randomized to receive or not pre-irradiation CT (sandwich CT), but all received RT and CT after RT, based on CCNU and VCR. Both studies showed no-statistically significant difference in outcomes (in GPO-MBL 80, 6-year EFS was 46% (18, 19); in SIOP II patients with AR-MB had 6-year EFS was 64.7% (RT alone) and 58.9% (sandwich CT); patients with HR-MB had 5-year EFS was 52.8% (no sandwich CT) Vs. 56.3% (sandwich CT) (26) (**Tables 1**, **3**, **4**). Simultaneously, the French M7 Cooperative Study (28) (1985-1988) combined pre-irradiation CT based on the “8-in-1” regimen and two cycles of high-dose MTX (in the average-risk group the MTX was given before RT, while in the high-risk group MTX was administered concomitantly to RT) followed by RT and, in the high-risk group only, followed by 4 more cycles of “8-in-1” CT. The M7 trial obtained 5-year DFS of 74% for patients with AR-MB and 57% for high-risk patients (28). At the same time, the POG 8695 (31) pilot study (1986-1990) found discouraging results when CT was administered before RT. There was a 43% of objective response rate and 23% of patients had progressive disease during CT, leaving a 2-year PFS of only 40% (31). Subsequently, the randomized POG 9031 (40) trial (1990-1996) compared outcomes of children treated with 3 cycles of CDDP and VP16 either before or after RT, with non-statistically significant difference between the treating arms (5-year EFS for pre-RT CT was 66% Vs. 70% for RT first) (40). Despite this, objective response rates were 66% for pre-RT CT and 86% for RT first (p=0.01) (40) (**Tables 3**, **4**).

The GPOH phase II pilot study HIT 88/89 (34) (1987-1991) and the MSFOP93 (74) study (1991-1998) examined the efficacy of pre-irradiation CT in children with AR-MB, achieving a 5-year PFS of 64.8% (34, 74). The results of the HIT 88/89 study led to the development of the randomized HIT 91 (42, 43) trial (1991-1997), which compared RT followed by maintenance CT Vs. the sandwich treatment scheme used in the earlier HIT 88/89 pilot study, with VCR administration concurrent with RT in all cases. In addition, patients with inadequate response after sandwich CT and RT also received maintenance CT. This trial confirmed that pre-irradiation CT and subsequent delay in RT administration had a negative impact on outcome in M0 patients (10-year EFS with sandwich CT Vs. post-irradiation CT was 53% and 83%, respectively) and in M1 patients (the 10-year EFS with sandwich CT Vs. maintenance CT was 36% and 71%, respectively), but, in contrast, the outcome was similar in M2/3 patients regardless of the treatment received (10-year EFS with sandwich Vs. maintenance CT was 40% and 32%, respectively) (42, 43) (**Tables 3**, **4**).

Simultaneously, the randomized SIOP/UKCCSG PNET3 (75) trial (1992-2000) compared, in non-metastatic patients, RT alone Vs. sandwich CT prior to RT with more intense CT regimen than the earlier SIOP trial, based on VCR, etoposide (VP16), CBDCA and CP. All M2/M3 patients received both RT and CT. This trial obtained opposite results to the previous ones in non-metastatic patients: pre-irradiation CT significantly improved EFS compared to RT alone (5-year EFS was 74.2%, while 5-year EFS for RT alone was 59.8%), without a non-statistically significant improvement in OS (5-year OS was 76.7% for pre-irradiation CT+RT and 64.9% for RT alone) (75). For M2/M3 patients, outcome rates were similar (5-year EFS of 34.7% (44)). The differences observed in non-metastatic patients could be explained by the absence of CT at any time in patients treated with early RT, which is known to be beneficial. Nevertheless, the EFS rates of the HIT 91 trial remain better than those achieved with the SIOP/UKCCSG PNET3 treatment plan. Concurrently, the French SFOP (45) study for metastatic MB (1993–1999) obtained a 5-year EFS of 49.8% alternating 8-in-1 cycles with CBDCA and VP16 before and after RT (45) (**Tables 3**, **4**).

These different trials have failed to confirm the benefit of pre-RT CT. On the contrary, there is concern regarding the risk of delaying RT secondary to the administration of CT. In this regard, the different trials show a 9.6-33% progression rate during CT treatment, prior to the initiation of RT (31, 37, 40, 42, 45, 46). At St. Jude Hospital they analyzed failure patterns in infants and children with MB treated with pre-RT CT from 1984 to 1993 and observed that the risk of neuroaxial progression increased with CT duration (104). Currently, it is recommended to administer CT 4 weeks after ending RT and, ideally, during RT.

### 4.13 Is chemotherapy concomitant to radiation therapy beneficial?

**Figure 11:**
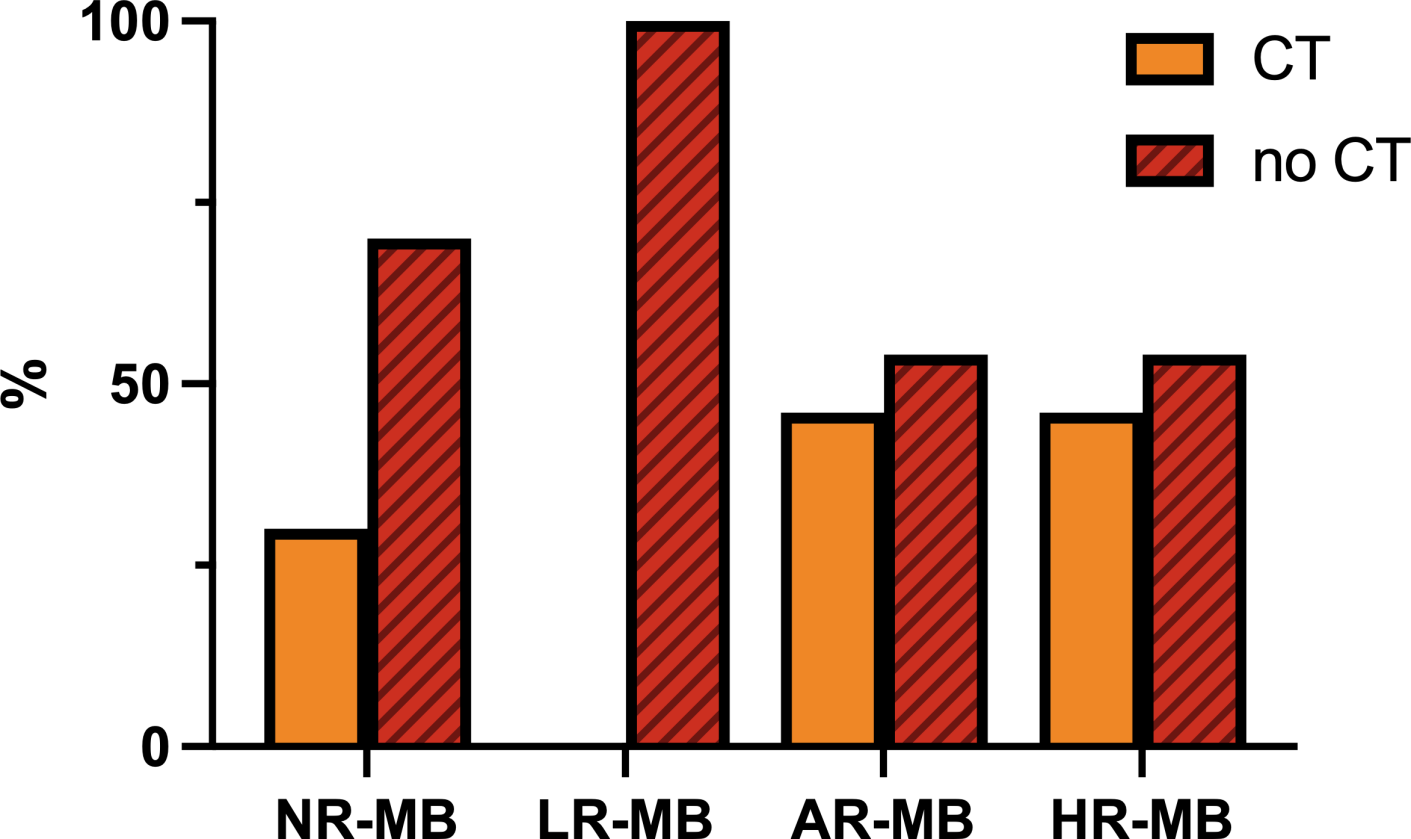
Frequency of administration of chemotherapy (CT) concomitant to radiation therapy, divided by risk. AR-MB, average-risk medulloblastoma; LR-MB, low-risk medulloblastoma; NR-MB, non-defined risk medulloblastoma; HR-MB, high-risk medulloblastoma.

CT plays an important role in enhancing the effect of RT. Various treatment protocols administer CT concomitant to RT, but the frequency varies depending on the risk classification (**Figure 11**). The most widely used drug is weekly VCR (SIOP I (16), CCG 942 (21), Philadelphia-Washington-Dallas (22, 24), CHP455 (72), CCG 9892 (73), HIT 91 (42, 43), PNET4 (81), CHP693 (82), COG ACNS0331 (83), among others). In an attempt to intensify treatment, daily CBDCA was used as a radiosensitizer together with weekly VCR during RT in the phase I/II study COG 99701 (53) (1998-2004) with promising results (5-year PFS of 59-71% (53)). This effect was subsequently tested in the trial ACNS0332 (7) (2007-2018), which randomized whether or not to add daily CBDCA to weekly VCR during RT. Overall, the trial showed no benefit (5-year EFS 66.4% with Vs. 59.2% without CBDCA, p=0.11) (7). The trial results showed exclusively a benefit in molecular group 3 (5-year EFS 73.2% with CBDCA Vs. 53.7% without CBDCA, p=0.047) (7). In the remaining molecular subgroups, it was even found to be detrimental in some molecular subgroups (e.g., Wnt and SHH) (7) (**Table 2**). Once the PNET5 study is concluded, it may finally be elucidated whether daily administration of CBDCA during RT is advisable or not. In any case, it is clear that the toxicity of weekly VCR during RT is lower than the one observed with daily CBDCA.

Other treatment schemes using other drugs include: the POG 9631 (52) trial (1998-2002), which administered a 3-week course of daily VP16 during RT with good survival rates and good tolerability once the daily dose was reduced to 35 mg/m2/day instead of the original 50 mg/m2/day (52); the MGH-99-271 (62) trial, in which patients received VCR, CBDCA, VCR+CBDCA, oral VP16 or no CT; and finally, the Japanese Pediatric Brain Tumor Consortium (1997-2006 and 2006-2014) (51), who used intrathecal MTX and intravenous VCR, CDDP, VP16 and CP with a promising 5-year PFS of 82.1% despite reducing CSI to 18 Gy (51) (**Tables 3**, **4**).

Except for the PNET5 study, there have been no studies focused on the randomization of CT during RT. There are no studies that exclusively evaluate the benefit of the addition of VCR during RT, so the effectiveness of its administration cannot be assured. Randomized studies conducted in the past (SIOP I (16), CCG 942 (21), Philadelphia-Washington-Dallas (22, 24)) randomize the administration of CT or no CT (concomitant VCR and maintenance CT), and all of them show a benefit of CT administration. However, the proportion of benefit due to concomitant VCR administration is unclear. The CCG 921 (32) trial randomized two regimens of CT: regimen A, which included VCR during RT followed by maintenance CT based on VCP regimen, and regimen B, which included two cycles of “8in1” CT before RT and eight cycles of “8in1” CT after RT, without any CT during RT. In this trial there was a benefit toward regimen A. Although this benefit could be justified in part by the increased dose intensity of VCR, it is more likely due to the earlier administration of RT. Similarly, the HIT 91 (42, 43) trial also showed a benefit of the arm including VCR during RT. However, it is likely that this benefit was secondary to the delay and higher rate of interruptions in RT administration in the other arm (**Tables 3**, **4**).

Weekly VCR primarily, but also daily CBDCA (alone or in combination of both), are frequently administered as radiosensitizers concomitant to RT. The specific benefit of their administration has not been explored in depth.

### 4.14 Does intensified chemotherapy with stem cell rescue have a role in medulloblastoma?

The use of intensified CT with stem cell rescue has also been tested with the aim of increasing survival. The multicenter SJMB 96 (49, 79) trial (1996-2003) combined a risk-adapted CSI dose with 4 cycles of dose-intensive CT based on VCR, CDDP and CP followed by stem cell rescue with good results (5-year EFS of 83% for localized AR-MB, 66% for M patients and 70% for patients with HR-MB) despite reducing total VCR and CDDP doses and treatment time compared to other established regimens (49, 79)). The SJMB03 (8) trial used the same CT scheme as its predecessor and obtained similar results (5-year PFS of 83.2% for patients with AR-MB and 58.7% for the HR-MB ones) (8) (**Tables 3**, **4**).

It was also tested in the Gustave Roussy (60) (2001-2010) and PNET HR+5 (67) (2009-2012) trials for high-risk patients. Both protocols used 2 cycles of CBDCA+VP16, followed by 2 cycles of high-dose thiotepa followed by stem cell rescue, RT and maintenance CT, with similar outcomes (5-year EFS of 65% for the French and 5-year PFS of 76% for the PNET HR+5) (60, 67) (**Table 4**).

It was also tested by the Koreans (56) (1999-2005), who used 1-2 cycles of high-dose CT with a combination of several drugs (CP + melphalan, CBDCA + thiotepa + VP16, or busulfan + melphalan) followed by stem cell rescue in high-risk patients aged 1-16 years old with similar outcomes (3-year EFS of 83% in older than 3 years old, and 3-year EFS of 62.5% in younger than 3) (**Table 4**).

In contrast, the SFOP (45) and the CCG 99702 (55) trials for patients with HR-MB obtained poorer survival rates (5-year EFS 49.8% and 46%, respectively), the latter due to a high-incidence of sinusoidal obstructive syndrome (45, 55). The Head Start II (50) (1997-2003) and III (63) (2003-2009) trials, included children younger than 10 years old and used myeloablative CT as consolidation of a previous induction CT. In the Head Start II (50), it failed to maintain a good survival rate despite excellent response rates observed with the induction CT (response rate to induction CT 91%; 3-year EFS of 49%) (50). The Head Start III trial (2003-2009) obtained average survival rates as a whole (5-year EFS of 46%), but very poor survival rates in 6-10 year old children (63) (**Table 4**).

Finally, the Japanese Pediatric Brain Tumor Consortium (51, 66) (1997-2006 and 2006-2014) obtained very good survival rates (3-year PFS 93.9% for patients with AR-MB and 5-year PFS of 82.1% for HR-MB) despite reducing neuraxis irradiation dose to 18 Gy by combining it with concomitant intrathecal MTX, intravenous CP, CDDP, VP16 and VCR and one cycle of thiotepa and melphalan followed by stem cell rescue (51, 66) (**Tables 3**, **4**). However, this trial had a small sample size, so the results should be interpreted carefully (99).

Despite good results in the past, schemes based on intensified CT or high-dose CT followed by autologous stem cell rescue are not widely used as they have not shown any real benefit compared to standard CT (49, 105). However, given the excellent survival rates reported by the Japanese (51, 66), this could be a matter of debate.

### 4.15 Is intrathecal chemotherapy beneficial in medulloblastoma?

Various regimens (GPO-MBL 80 (18, 19), Italy 1985 (69), CNS-85 (30), Japan 1997 (51), Japan 2006 (66)) include intrathecal/intraventricular administration of MTX (**Tables 1**, **3**, **4**). Its administration is even more frequent in radiation-sparing treatment protocols for infants with MB (3, 4). The Italian study is the only one to randomize its administration, and concludes that it is not advisable because it does not improve survival and worsens the cognitive outcome of these patients (69). On the contrary, children treated with CT and intraventricular MTX according to the (MET-)HIT-2000-BIS4 study showed similar IQ scores compared to children with ependymoma treated with focal RT (106). In the Japanese Pediatric Brain Tumor Consortium studies, intrathecal MTX administration was safe but, despite having excellent survival rates, it is not possible to determine the contribution of intrathecal MTX to these results. They believe that its administration allows to safely decrease the CSI dose to 18 Gy (51, 66).

### 4.16 Are there any other treatments useful in medulloblastoma?

The randomized trial ACNS0332 (7), based on experience with neuroblastoma, also tested the survival-enhancing potential of isotretinoin as a proapoptotic agent. This randomization was closed in 2005 due to the absence of a significant effect (5-year EFS of 68.6% with isotretinoin Vs. 67.8% without it) (7).

Other novel therapies, including targeted treatments such as vismodegib, are currently being investigated in the SJMB12 trial (NCT01878617, from 2013 to present). The efficacy of this approach remains unknown to date, but it is essential to move forward and include these new targeted therapies.

### 4.17 What are the main side effects of the treatment? Have we improved them over time?

Long-term survivors of MB suffer neurological deficits, hearing loss, endocrine deficits, growth restriction, cognitive impairment, increased risk of stroke, social deficits and impaired quality of life, among others (107, 108).

CSI is known to be a major cause of cognitive impairment. This deterioration is inversely related to the age of the patient, having a great impact in patients younger than 7-10 years old (69, 106, 109). It is also directly related to RT dose and is progressive over several years after treatment (69, 109, 110). However, reducing the RT dose does not provide the decrease in side effects that might be expected. Analysis of the intellectual outcome of children treated with the CCG A9961 trial found that the intellectual decline observed in children receiving CSI with 23.4 Gy was similar to that of previous studies administering 36 Gy (111). Conversely, the SJMB 96 trial did show that patients receiving 23.4 Gy had less cognitive impairment than patients receiving 36-39.6 Gy, and it was especially important in children younger than 7 years old (112). Patients treated with HFRT had better verbal activity in children younger than 8, but, on the contrary, had worse growth, and had no other significant differences observed compared to patients treated with conventional RT (113).

Intrathecal MTX was shown to have a detrimental effect on cognitive function by the Italian group, again especially in children younger than 10 years old, without an improvement in survival (69), but this is opposite to the results shown in the (MET-)HIT-2000-BIS4 trial (106).

Patients included in the SIOP/UKCCSG PNET3 study who received CT prior RT were found to have worse health status, poorer quality of life and more behavioral and emotional problems compared to patients who received RT alone (114). However, this study showed that patients with CT+RT had a significantly better EFS than those treated exclusively with RT (75), so these side effects may not be so bad after all.

Similarly, some of the CT drugs used in the treatment of MB have well-known intrinsic side effects and dose reductions are often needed due to toxicity, especially in older patients. Dose reductions of VCR or CDDP do not seem to affect survival (115, 116), so it might be advisable to use lower doses in future trials. Regarding CCDP-induced hearing loss, amifostine has been applied with positive results, especially in patients with AR-MB (117).

But not all the side effects are treatment-related. Tumor location is also important. Tumors arising in the vermis induce emotional disturbances whereas hemispheric tumors cause cognitive deterioration (69). Moreover, different molecular subgroups have different toxicities (118, 119). Other risk factors for further neurocognitive impairment include post-operative mutism and higher intelligence at diagnosis (110).

The improvement in treatment-related toxicity has been small. Despite reducing RT dose, despite avoiding some drugs, despite applying prophylactic treatments and despite technological advances, treatment-related toxicity remains significant and most long-term survivors experience side effects. Cognitive and behavioral therapies have been applied with positive results, especially in young children, and should be applied more widely (120, 121). Other interventions have been proven ineffective (122).

## 5 Conclusions

The treatment of MB has improved significantly over time. The improvement was big from 1950s to 1970s with the introduction of RT and CT. Since then, the improvement has been smaller. The results of the different studies and trials performed, even if they have yielded negative results, have been the basis for reaching the survival rates we are achieving today. Among the various studies performed, some are worth highlighting. Firstly, those performed at the Royal Marsden Hospital (11), which demonstrated the benefit of adding CT to the treatment of MB. In addition, the San Francisco (9) treatment protocol (1966–1987) obtained impressive survival rates (5-year OS 91%) (9). Similarly, the Japanese have obtained excellent survival rates that deserve attention (51, 66). Some other studies (25, 38, 42, 70) have shown that reduction of treatment intensity should be done with cautioun to avoid poor outcomes. Furthermore, the COG ACNS0331 trial has shown that it is feasible to administer RT boost exclusively to the tumor bed instead of the whole posterior fossa (83). Finally, the SJMB03 trial has proven the prognostic implications of molecular subgrouping in MB (8).

Children should be treated in experimented centers, where they should receive the best treatment known to date and with the most modern technological means. Adequate management of MB in children >3 years old consists of:

1. First, surgery, with maximum safe resection but without significant secondary neurological deficits;
2. Secondly, RT. It should be administered early (ideally within the first 28 days after surgery), craniospinal, with boost to the tumor bed, over 6 weeks, without interruption, and probably with concomitant CT. Proton or photon therapies are acceptable, provided that modern photon techniques such as IMRT are used.
3. Thirdly, CT. A good option is to administer 6-9 cycles of a combination of CDDP, CCNU, VCR and CP every 4-8 weeks.
4. Fourthly, we should consider introducing targeted therapy, such as SHH pathway inhibitors, as front-line therapy.
5. Finally, we should also implement the use of therapies known to reduce side effects, such as cognitive and behavioral therapies and amifostine.

It is crucial to continue international collaboration so that studies rapidly include a sufficient number of patients to be able to draw conclusions quickly. The development of clinical trials involves major effort. Parents of children participate generously in studies to advance knowledge. However, some studies are communicating their results more than 10 years after their closure so it is impossible to benefit from their results. It is critical to communicate the results as soon as possible and to avoid repeating what has already been demonstrated.

The next step now is to further emphasize the importance of risk classification according to molecular features. We should keep trying to adjust treatment to reduce treatment-related side effects, but without decreasing survival. However, we must be careful because the worst side effect is disease progression and death.

## Data availability statement

The original contributions presented in the study are included in the article/supplementary material. Further inquiries can be directed to the corresponding author.

## Author contributions

MO-M, BL-I, and ÁT provided concept, design, and manuscript preparation. MO-M and LM-L performed the search and read the titles and abstracts of all the papers that met the criteria. MO-M deeply reviewed the selected papers and wrote the first draft. All authors contributed to the article and approved the submitted version.
